# Relativity and decoherence of spacetime superpositions

**DOI:** 10.1038/s41534-026-01234-x

**Published:** 2026-05-13

**Authors:** Joshua Foo, Cendikiawan Suryaatmadja, Robert B. Mann, Magdalena Zych

**Affiliations:** 1 Centre for Quantum Computation & Communication Technology, School of Mathematics & Physics, The University of Queensland, St. Lucia, QLD Australia; 2 Department of Physics, Stevens Institute of Technology, Hoboken, NJ USA; 3 Department of Physics and Astronomy, University of Waterloo, Waterloo, ON Canada; 4 Department of Physics, Kyushu University, 819-0395, 744 Motooka, Fukuoka, Japan; 5 Quantum and Spacetime Research Institute, Kyushu University, 819-0395, 744 Motooka, Fukuoka, Japan; 6 Institute for Advanced Study, Kyushu University, 819-0395, 744 Motooka, Fukuoka, Japan; 7 Perimeter Institute, Waterloo, ON Canada; 8 Department of Physics, Stockholm University, AlbaNova University Center, Stockholm, Sweden; 9 Centre for Engineered Quantum Systems, School of Mathematics and Physics, The University of Queensland, St. Lucia, QLD Australia

**Keywords:** Quantum information, Quantum mechanics, Theoretical physics

## Abstract

It is univocally anticipated that in a theory of quantum gravity, there exist quantum superpositions of semiclassical states of spacetime geometry. Such states could arise, for example, from a source mass in a superposition of spatial configurations. In this paper, we introduce a framework for describing such “quantum superpositions of spacetime states.” We introduce the notion of the relativity of spacetime superpositions, demonstrating that for states in which the superposed amplitudes differ by a coordinate transformation, it is always possible to re-express the scenario in terms of dynamics on a single, fixed background. Our result unveils an inherent ambiguity in labelling such superpositions as genuinely quantum-gravitational, which has been done extensively in the literature, most notably with reference to recent proposals to test gravitationally-induced entanglement. We apply our framework to the above-mentioned scenarios, looking at gravitationally-induced entanglement, the problem of decoherence of gravitational sources, and clarifying commonly overlooked assumptions. In the context of decoherence of gravitational sources, our result implies that the resulting decoherence is not fundamental, but depends on the existence of external systems that define a relative set of coordinates through which the notion of spatial superposition obtains physical meaning.

## Introduction

The twin discoveries of quantum mechanics and Einstein’s theory of general relativity revolutionized our understanding of reality. Perhaps the most distinctive features of these respective theories are the superposition principle and the notion of curved spacetime. The former postulates that a quantum system with a distinct set of possible configurations may also exist in a state featuring many of these configurations “at once.” The latter was Einstein’s geometric interpretation of gravity as the curvature of a pseudo-Riemannian manifold, generated by the presence of mass and energy^1^.

One of the most significant challenges for modern physicists is finding a consistent unification of quantum theory with general relativity. Existing attempts can be split into background-dependent approaches–including perturbative quantum general relativity and string theory–and background-independent approaches–including causal set theory and loop quantum gravity. Effective field theory (EFT) approaches^2,3^ have also been developed and successfully applied to the description of spin-2 gravitons at low energies (and the resulting emergence of classical general relativity in this limit)^4–9^.

Most theories of quantum gravity allow for the possibility of quantum superpositions of gravitational fields or spacetime geometries^10^. Taking the foundational principles mentioned above, this is a simple consequence of applying the superposition principle to semiclassical curved spacetimes (i.e., solutions to Einstein’s equations peaked around a definite, classical value in the gravitational phase space). In other words, despite the various technical challenges in writing down a theory combining relativistic gravitation with quantum mechanics, it is nevertheless univocally anticipated that such a theory must be able to describe spacetime as possessing quantum-mechanical degrees of freedom, whose states reside in a complex Hilbert space and may be placed in quantum superpositions of different configurations. Background-independent approaches such as the Wheeler-DeWitt equation^11,12^ and loop quantum gravity^13–15^, which motivate the framework developed here, allow for such scenarios to be studied^16–21^.

In this article, without relying on a particular background-independent formulation of quantum gravity, we take a foundational, “bottom-up” approach to the question of the physical meaning of “superpositions of spacetime metrics” (or superpositions of Newtonian gravitational fields in the nonrelativistic limit). We mainly focus on the spatial superposition of a source mass, but our approach and main results hold for superpositions of states related by any diffeomorphism. This includes, but is not limited to, transformations corresponding to symmetries of dynamics (e.g., translations, rotations). More generally, our results rely on the coordinate invariance of quantum theory and the linearity of quantum superpositions, and thus apply to arbitrary coordinate transformations, irrespective of whether they correspond to conserved quantities or leave the action invariant.

Spatial superposition states are considered in the proposals for possible observations of gravitationally-mediated entanglement^22,23^, in the context of decoherence that they may induce on quantum systems^24–29^, and the indefinite causal structures they give rise to^30–34^. Here we demonstrate that any effects emerging due to a spatial superposition of a massive object can be formally reproduced using a single spacetime metric, due in part to two basic tenets of quantum mechanics and relativity: linearity of quantum theory and invariance of dynamics under general coordinate transformations in general relativity^35^. We show how these principles combined lead to the notion of the “relativity” of spacetime superpositions (RSTS) and to invariance of probabilities in quantum mechanics under quantum transformations between classical coordinates, i.e., transformations between sets of coordinates associated with different amplitudes of a system (which may include spacetime plus matter DoFs) in a superposition. This is different from but is conceptually related to the approach of quantum reference frames (QRFs). However, unlike QRFs^36–41^, which associate reference frames with quantum systems (and generalizations thereof to gravitating systems^38,39,42^) and transformations between them, we address the different problem of the role that passive relabeling of coordinates has on the interpretation of semiclassical spacetimes in quantum superposition, including dynamics of other systems residing within.

As we have alluded, the principle of RSTS is motivated by and related to the notion of background independence in general relativity and, by extension, canonical approaches to quantum gravity, including the above-mentioned Wheeler-DeWitt equation and loop quantum gravity. In such approaches, one starts with the Hamiltonian (ADM) formalism of general relativity, chooses canonical variables (in the Wheeler-DeWitt equation, these are the spatial 3-metric *h*_*ij*_ and the extrinsic curvature *K*_*ij*_), and quantizes them à la Dirac^43^. This leads to the Hamiltonian and diffeomorphism constraints on the space of physical states, the latter imposing the diffeomorphism invariance of such states. This is also an assumption underlying the RSTS framework developed in this article, namely that superpositions of geometries related by a diffeomorphism–such as configurations differing only by a coordinate translation–should be considered physically indistinct in the absence of external reference systems that break this symmetry. While this assumption differs from the usual intuition acquired from textbook nonrelativistic quantum mechanics, as we discuss in the paragraph on “Background Independence” in “Preliminaries: Relativity of Superpositions“, it is entirely reasonable that a quantum theory of general relativity should have at its foundation this principle.

Returning to the examples mentioned above^22–34^, there have been many claims that such scenarios represent genuine examples of “quantum-gravitational spacetime” ^44–49^ (quantum-gravitational insofar as such solutions cannot be described using the frameworks provided by quantum theory and general relativity), or in the nonrelativistic limit, superpositions of Newtonian gravitational fields^50–59^. However, the conclusion reached via RSTS is that this is not unambiguously true. Just as for classical configurations, where only relative distances between objects are of physical significance, the same is also true for gravitational sources in a superposition of different configurations, where these configurations are related by a coordinate transformation^60^.

To understand the impact of the above result, we apply it in four distinct contexts of recent interest: gravitationally-induced entanglement^22,23,44^, decoherence of spatial superpositions of black holes^25,26^, and in the Appendices, decoherence of dark matter^27^, and the gedanken experiment in Ref. ^61^. We discuss the implications of our findings for the conclusions that can be drawn from such scenarios and possibilities for lifting the inherent ambiguities by considering superpositions of non-diffeomorphic metrics.

## Results

### Preliminaries: Relativity Of Superpositions

Before discussing superpositions of gravitating quantum systems, it is important to clarify the key concepts relevant to that discussion in the general context of superpositions of quantum states, without including any interactions.

General covariance demands that absolute position has no physical meaning^62^. For example, we may consider a scenario with two particles, one localized at some position *x* and another at some translated position labeled as *x* + *X*. In any translationally invariant theory, this scenario is equivalent to the scenario in which the first particle is assigned a position *x* − *X* and the second one is assigned a position *x*. Any difference between the two scenarios is merely apparent, attributed to a choice of a coordinate system, which emphasises that only relative distances are physically meaningful, with coordinate systems playing a subordinate, rather than fundamental role.

We now outline why this idea, despite intuition, does extend to quantum theory, which we then present rigorously in the next section in the context of superpositions of “semiclassical” (i.e., coherent) states of spacetime.

Consider a scenario with two particles, where the first is assigned a state $\left|x\right\rangle$ corresponding to a position *x*, while the second is in a superposition of positions,

$$\frac{1}{\sqrt{2}}(\left|x+{X}_{1}\right\rangle +\left|x+{X}_{2}\right\rangle ),$$

where 〈*x* + *X*_1_∣*x* + *X*_2_〉 = 0. If indeed only relative distances are physically meaningful in describing the configuration of the particles, then the joint state

1 $$\frac{1}{\sqrt{2}}\left|{x}_{1}\right\rangle \otimes (\left|{x}_{2}+{X}_{1}\right\rangle +\left|{x}_{2}+{X}_{2}\right\rangle )$$

would be equivalent to a seemingly different state

2 $$\frac{1}{\sqrt{2}}(\left|{x}_{1}-{X}_{1}\right\rangle +\left|{x}_{1}-{X}_{2}\right\rangle )\otimes \left|{x}_{2}\right\rangle$$

in which the second particle is localized at a position *x*, while the first is in a superposition of translations. For each individual amplitude, the two states can be related by a passive translation, represented as a unitary operator

$$\widehat{T}({X}_{i})\equiv {\widehat{T}}_{1}({X}_{i})\otimes {\widehat{T}}_{2}({X}_{i})$$

where *i* = 1, 2, that enacts a change of classical coordinates labelling the positions, and thus relabelling the basis states of all the systems (here, the two particles):

3 $$\left|{x}_{1}\right\rangle \otimes \left|{x}_{2}+{X}_{i}\right\rangle =\widehat{T}({X}_{i})\left|{x}_{1}-{X}_{i}\right\rangle \otimes \left|{x}_{2}\right\rangle .$$

The existence of such a transformation is a consequence of the linearity of quantum mechanics i.e. Wigner’s theorem^63^. Importantly, a mapping between the superposition states, denoted generically by the operator $\widehat{{\mathcal{T}}}$, also exists:

4 $$\begin{array}{l}\frac{1}{\sqrt{2}}\left|{x}_{1}\right\rangle (\left|{x}_{2}+{X}_{1}\right\rangle +\left|{x}_{2}+{X}_{2}\right\rangle )\\ \,=\frac{\widehat{{\mathcal{T}}}}{\sqrt{2}}(\left|{x}_{1}-{X}_{1}\right\rangle +\left|{x}_{1}-{X}_{2}\right\rangle )\left|{x}_{2}\right\rangle ,\end{array}$$

where $\widehat{{\mathcal{T}}}$ in the present case takes the particularly simple form,

$$\widehat{{\mathcal{T}}}=\mathop{\sum }\limits_{i=1,2}\widehat{T}({X}_{i})(\left|{x}_{1}-{X}_{i}\right\rangle \left\langle {x}_{1}-{X}_{i}\right|),$$

which is essentially an application of the translation operator $\widehat{T}({X}_{i})$, quantum-controlled on the basis states associated with particle 1. While it is uncontroversial that such an operator $\widehat{{\mathcal{T}}}$ exists and can be seen as a change of basis in the two-particle Hilbert space, we argue in the next section that it can also be seen as a quantum change of coordinates, to coordinates that are “in a superposition” of different translations relative to the original coordinate system.

**Fig. 1:**
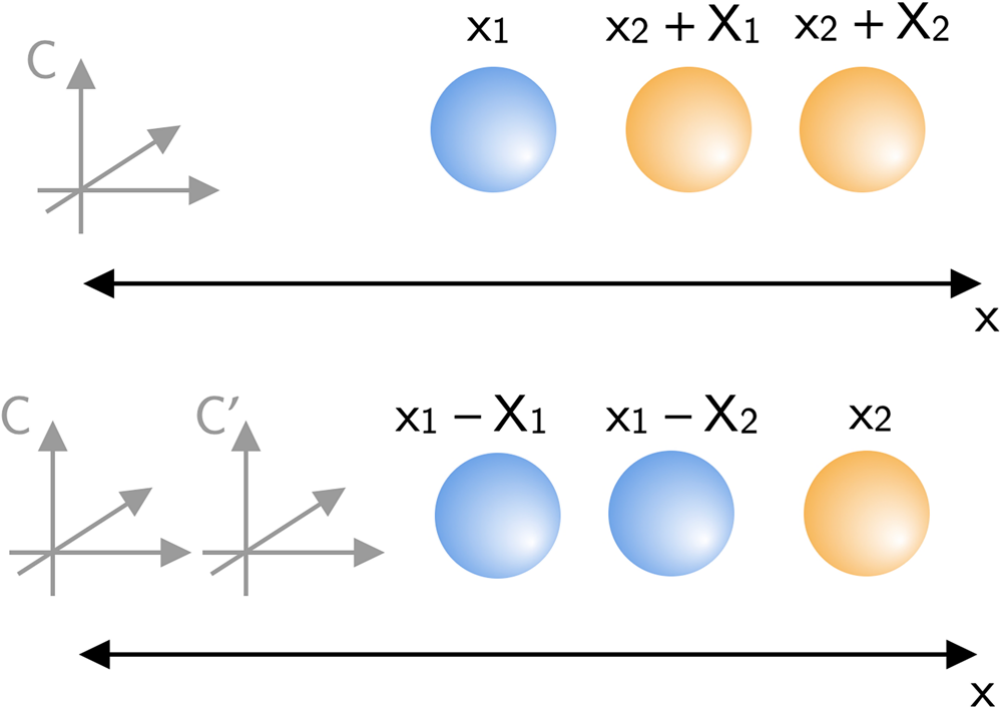
Schematic diagram illustrating the states Eqs. (1) and (2) using two sets of quantum coordinates. In the top panel, particle 1 is a fixed distance *x*_1_ from the origin of coordinates (denoted *C*), while particle 2 is in a superposition of two positions *x*_2_ + *X*_1_, *x*_2_ + *X*_2_. In the bottom panel, the coordinate system is “in superposition” relative to *C* displayed in the top panel, but relative distances between the two particles are preserved in each branch.

We stress that what Eq. (4) represents is that the choice and labelling of basis states for a quantum system is merely conventional, and we recognise that this freedom to relabel the basis is represented by a unitary mapping between different bases, even if no classical coordinate transformation exists that such a unitary represents. We sketch an example of the above-discussed interpretation of the unitary implied by Eq. (4) as a transformation to coordinates in a “superposition” of translations in Fig. 1, for the case of two particles in an entangled state and separated by the same distance *X* in each amplitude.

### Preliminaries: States, Diffeomorphisms, Background Independence

#### Hilbert Space And Semiclassical Spacetime States

In dealing with quantum superpositions of spacetime and dynamics of additional DoFs residing within such spacetimes, we adopt standard assumptions used in quantum gravity phenomenology. In particular:

1. The gravitational field can be placed in a superposition of “semiclassical” states, $\left|g(x)\right\rangle ,\left|g(y)\right\rangle$, each corresponding to coherent states of the metric parametrized by coordinates *x*, *y* and associated with a classical solution to Einstein’s field equations for appropriate matter distributions.
2. The dynamics of other DoFs in the presence of the superposition of spacetimes can be described by quantum-controlled dynamics: in each of the amplitudes of the superposition, the dynamics are described by quantum theory in the spacetime of that amplitude.
3. The Hilbert space of the combined spacetime and system DoFs can be described as the tensor product ${\mathcal{H}}={{\mathcal{H}}}_{{\rm{S}}}\otimes {{\mathcal{H}}}_{{\rm{DoF}}}$ where ${{\mathcal{H}}}_{{\rm{S}}}$ is the Hilbert space associated with the spacetime degrees of freedom and, depending on the context, ${{\mathcal{H}}}_{{\rm{DoF}}}$ can be further decomposed as a product of different matter and/or field DoFs (for example when considering the light-matter interaction, see “Coherence of Spacetime Superpositions”).

#### Unitary Representation Of Diffeomorphisms

A (global) diffeomorphism between the manifolds $M,{M}^{{\prime} }$ is a smooth map $\varphi :M\mapsto {M}^{{\prime} }$ with a smooth inverse map *φ*^−1^. The manifolds $M,{M}^{{\prime} }$ represent the same geometry and are therefore physically indistinguishable. To emphasise this, we denote two diffeomorphically related manifolds, each endowed with a metric *g*(*x*), *g*(*y*) with the same “*g*” but parametrised by different coordinate systems *x*, *y*. The coordinates *x*, *y* are associated with the respective configurations of the source mass, and could describe, for example, coordinates centered at the position of the mass. Diffeomorphisms in general relativity correspond to coordinate transformations that relate different descriptions of the same physical situation. These transformations act passively on the coordinates; when considering the corresponding transformations on the quantum state, their effect is represented by unitary operators, consistent with Wigner’s Theorem^64^.

Therefore, the semiclassical configurations associated with the states $\left|g(x)\right\rangle ,\left|g(y)\right\rangle$ are related via the action of the unitary operator $\widehat{T}(x,y)$,

5 $$\left|g(x)\right\rangle =\widehat{T}(x,y)\left|g(y)\right\rangle$$

and its inverse,

6 $$\left|g(y)\right\rangle ={\widehat{T}}^{\dagger }(x,y)\left|g(x)\right\rangle$$

where $\widehat{T}(x,y)$ could depend on some potentially complicated function of the spacetime coordinates that characterises the distance between the points in *x*, *y* respectively. As an illustration, consider the case where $\left|g(x)\right\rangle ,\left|g(y)\right\rangle$ are related by a translation of the coordinates *X*. Then, we can write

$$\left|g(x)\right\rangle =\widehat{T}(X)\left|g(y)\right\rangle .$$

#### Initial and Final States

**Fig. 2:**
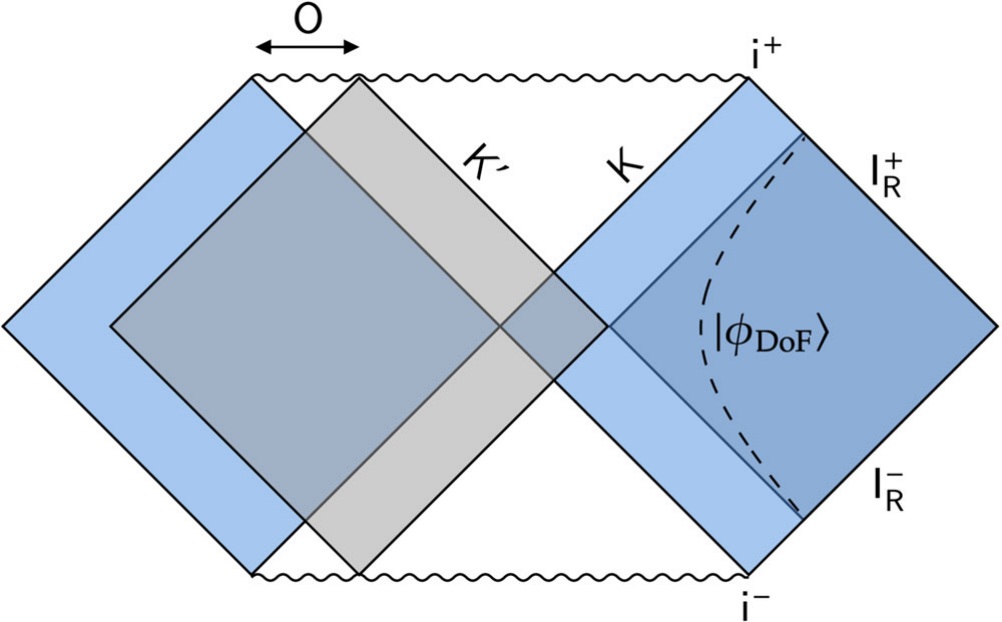
An example of Eq. (7). Two Kruskal wedges, *K* and ${K}^{{\prime} }$, are in a superposition of the Kruskal coordinate *X* through distance *O*. A quantum system follows the dashed worldline in both branches of the superposition; in ${K}^{{\prime} }$, this corresponds to a fixed radial position above the event horizon, while in *K*, it is non-stationary.

Throughout this paper, we will be interested in the dynamics of quantum systems residing within the diffeomorphically related spacetime superpositions discussed so far. For example, we will analyze initial states of the form,

7 $$\frac{1}{\sqrt{2}}(\left|g(x)\right\rangle +\left|g(y)\right\rangle )\otimes \left|{\phi }_{{\rm{DoF}}}\right\rangle$$

describing the case where the state of the additional DoFs $\left|{\phi }_{{\rm{DoF}}}\right\rangle$ factors from the state of the metric. Tacitly, $\left|{\phi }_{{\rm{DoF}}}\right\rangle =\left|{\phi }_{{\rm{DoF}}}(x)\right\rangle =\left|{\phi }_{{\rm{DoF}}}(y)\right\rangle$, where our notation emphasizes the commonality of the state in both branches of the spacetime (we adopt this notational convention henceforth in scenarios where ambiguity does not arise). Let us remark that there is no unique meaning to the designation that *ϕ*_DoF_ are “common” across branches of spacetimes in superposition. In the language of Ref. ^42^, which we also discuss in the Discussion as a comparison with our framework, we are implicitly using the identity as a counterpart relation or comparison map. Without delving into the technical details (summarized in the Discussion), the counterpart relation endows meaning to the statement that two configurations (in Ref. ^42^, models *φ* = (*M*, *g*, *ψ*_DoF_) comprised of a manifold *M*, Lorentzian metric *g*, and additional DoFs *ψ*_DoF_) are “the same” across branches. Such a scenario has also been considered in, for example, Refs. ^65,66^, as well as in our Superposition of Geometries in the Gravitationally-Induced Entanglement Proposals, where we show that the claimed fundamental decoherence of black holes (i.e., due to their own Hawking radiation, rather than an “external” environment) actually results from a tacitly assumed “detector” that gains which-way information about the black hole’s location through its relative distance to the horizon. A pictorial example of another scenario that Eq. (7) could describe is shown in Fig. 2.

In addition, we will consider entangled states

$$\frac{1}{\sqrt{2}}(\left|g(x),\phi (x)\right\rangle +\left|g(y),\phi (y)\right\rangle )$$

where the shorthand notation $\left|g(\cdot ),\phi (\cdot )\right\rangle \equiv \left|g(\cdot )\right\rangle \left|{\phi }_{{\rm{DoF}}}(\cdot )\right\rangle \equiv \left|g(\cdot )\right\rangle \otimes \left|{\phi }_{{\rm{DoF}}}(\cdot )\right\rangle$ is implied.

In certain cases, we will examine a specific interaction between the additional DoFs, such as that of a two-level system (modelling some matter DoFs) with a quantum field, for which we employ the explicit notation,

$$\left|{\phi }_{{\rm{DoF}}}\right\rangle =\left|{\phi }_{{\rm{M}}}\right\rangle \left|{\phi }_{{\rm{F}}}\right\rangle .$$

To denote the final state of all involved systems, we use the state vector $\left|\Omega \right\rangle$. In some contexts we also look at conditional states given a measurement on only some subsystems. In this case we use the same subscripts as for initial states to label the different DoFs.

#### Unitary Transformations of Quantum Coordinates Acting on Multiple DoFs

We are interested in scenarios combining the concepts previously discussed. For example, consider the initial state Eq. (7). As stated in Eq. (5), the operator $\widehat{T}(x,y)$ will act on the coordinates of all the systems, which here means both the metric *and* any additional DoFs residing within. Implicitly then, $\widehat{T}(x,y)\equiv {\widehat{T}}_{{\rm{G}}}(x,y)\otimes {\widehat{T}}_{{\rm{DoF}}}(x,y)$ where ${\widehat{T}}_{{\rm{G}}}(x,y)$ acts on the metric Hilbert space. As such, Eq. (7) may be equivalently represented as (dropping the normalisation factor for simplicity),

8 $$\begin{array}{l}\left|g(x)\right\rangle \left|{\phi }_{{\rm{DoF}}}\right\rangle +{\widehat{T}}^{\dagger }(x,y)\left|g(x)\right\rangle \left|{\widehat{T}}_{{\rm{DoF}}}(x,y){\phi }_{{\rm{DoF}}}\right\rangle \\ \,=\left|g(x)\right\rangle \left|{\phi }_{{\rm{DoF}}}\right\rangle +{\widehat{T}}_{{\rm{G}}}^{\dagger }\left|g(x)\right\rangle \otimes {\widehat{T}}_{{\rm{DoF}}}^{\dagger }\left|{\widehat{T}}_{{\rm{DoF}}}{\phi }_{{\rm{DoF}}}\right\rangle \end{array}$$

having dropped the coordinates (*x*, *y*) in ${\widehat{T}}_{{\rm{G}}}^{\dagger }\otimes {\widehat{T}}_{{\rm{DoF}}}^{\dagger }$ in the second line for simplicity. We have used the notation $\left|{\widehat{T}}_{{\rm{DoF}}}{\phi }_{{\rm{DoF}}}\right\rangle \equiv {\widehat{T}}_{{\rm{DoF}}}\left|{\phi }_{{\rm{DoF}}}\right\rangle$ to denote the inverse of the transformation ${\widehat{T}}_{{\rm{DoF}}}^{\dagger }$, such that

$${\widehat{T}}_{{\rm{DoF}}}^{\dagger }\left|{\widehat{T}}_{{\rm{DoF}}}{\phi }_{{\rm{DoF}}}\right\rangle =\left|{\phi }_{{\rm{DoF}}}\right\rangle .$$

#### Background Independence

As we alluded to in the Introduction, an additional assumption of our framework, which follows from the principle of background independence in general relativity (and canonical approaches to quantum gravity), is that semiclassical spacetimes related by a diffeomorphism are physically equivalent. This notion stands in contrast with the intuition of non-relativistic quantum mechanics (NRQM), which is explicitly *background dependent*. In NRQM, “spacetime” itself is treated as a fixed, non-dynamical stage: spatial coordinates label absolute points that are physically distinguishable, even though the theory admits translation invariance at the dynamical level. Consequently, superpositions such as $(\left|x\right\rangle +\left|x+X\right\rangle )/\sqrt{2}$ are not simply equivalent to $\left|x\right\rangle$ or $\left|x+X\right\rangle$ alone. In background-independent theories, physical states must be invariant under diffeomorphisms and hence those that differ only by coordinate transformations are identified as physically equivalent. One consequence of such a principle is investigated in Superposition of Geometries in the Gravitationally-Induced Entanglement Proposals in the context of decoherence of spacetime superpositions. Specifically, a source mass that is in a superposition of different configurations, where all additional DoFs (e.g., matter fields, nonrelativistic quantum systems) are perfectly correlated with the configuration, is physically indistinguishable from the scenario in which the spacetime and additional DoFs are in a classically fixed state.

The different foundations of NRQM and background-independent theories of quantum gravity (and by extension, RSTS) calls for a justification of the latter. Many investigations on the foundations of quantum gravity have addressed this question^67–69^. Let us summarize some of the key points.

1. *Background independence reflects the diffeomorphism invariance of general relativity*. As discussed, the physical content of general relativity lies in the equivalence class of geometries related by coordinate transformations, not in any particular metric defined on a fixed background manifold. It is reasonable that a quantum theory of general relativity should retain this relational structure at the quantum level, meaning that geometries differing only by a diffeomorphism are physically indistinct.
2. *Background independence avoids privileging any specific spacetime geometry*. Background-dependent approaches operate by fixing a background spacetime and quantizing additional structures (e.g., gravitational waves or strings) based on this prior specification. However, in a full quantum theory, we might reasonably expect that geometry should emerge from the dynamics of quantum degrees of freedom. Background independence ensures that no particular geometry is built into the theory, allowing for the possibility that geometry emerges dynamically.
3. *Background independence underlies several core approaches to quantum gravity*. We have mentioned how canonical approaches like the Wheeler-DeWitt equation and loop quantum gravity enforce constraints (Hamiltonian and diffeomorphism) that remove dependence on a fixed coordinate system or background structure. The successes of these approaches^70–78^ lend credence to the strength of background independence as a fundamental physical principle.

### Preliminaries: Examples

#### Post-Newtonian Metric in Isotropic Coordinates

As a concrete application of the ideas presented above, consider the post-Newtonian expansion of the Schwarzschild metric in isotropic coordinates (*t*, *ρ*, *θ*, *ϕ*),

9 $$\begin{array}{rcl}{\rm{d}}{s}^{2} & = & -\left(1+\frac{2\Phi ({\rho }^{{\prime} })}{{c}^{2}}\right){\rm{d}}{t}^{2}\\ & + & \left(1-\frac{2\Phi ({\rho }^{{\prime} })}{{c}^{2}}\right)\left({\rm{d}}{\rho }^{2}+{\rho }^{2}{\rm{d}}{\Omega }^{2}\right)\end{array}$$

where ${\rm{d}}{\Omega }^{2}={\rm{d}}{\theta }^{2}+{\sin }^{2}(\theta ){\rm{d}}{\phi }^{2}$, $\Phi \equiv \Phi ({\rho }^{{\prime} })$ is the gravitational potential where ${\rho }^{{\prime} }=\sqrt{{\rho }^{2}+{\rho }_{0}^{2}-2\rho {\rho }_{0}\cos (\theta )}$ and *ρ*_0_ is the magnitude of the source mass displacement in the *z*-direction. One might suppose that the source mass is located in a superposition of the following coordinates relative to a test particle,

$$(t,{\rho }_{0},{\theta }_{0},{\phi }_{0}),(t,{\rho }_{0}^{{\prime} },{\theta }_{0},{\phi }_{0})$$

and so one can write the joint state as (up to normalisation),

10 $$(\left|g({\rho }_{0})\right\rangle +\left|g({\rho }_{0}^{{\prime} })\right\rangle )\otimes \left|{\phi }_{{\rm{test}}}\right\rangle$$

Since ${\rho }_{0},{\rho }_{0}^{{\prime} }$ are related by a coordinate transformation generated by the unitary ${\widehat{T}}^{\dagger }$ i.e. $\left|g({\rho }_{0}^{{\prime} })\right\rangle ={\widehat{T}}_{{\rm{G}}}^{\dagger }({\rho }_{0},{\rho }_{0}^{{\prime} })\left|g({\rho }_{0})\right\rangle$, one can write this state in the form,

11 $$\left|g({\rho }_{0})\right\rangle \left|{\phi }_{{\rm{test}}}\right\rangle +{\widehat{T}}_{{\rm{G}}}^{\dagger }\left|g({\rho }_{0})\right\rangle \otimes {\widehat{T}}_{{\rm{test}}}^{\dagger }\left|{\widehat{T}}_{{\rm{test}}}{\phi }_{{\rm{test}}}\right\rangle$$

in a similar manner to Eq. (8).

#### De Sitter Spacetime in Static Coordinates

As another example, let us consider de Sitter spacetime, described by the hyperboloid,

12 $$-{Z}_{0}^{2}+{Z}_{1}^{2}+{Z}_{2}^{2}+{Z}_{3}^{2}+{Z}_{4}^{2}={a}^{2}$$

where $a=\sqrt{3 /\Lambda}$ with *Λ* the cosmological constant, embedded in a flat five-dimensional Minkowski spacetime,

13 $${\rm{d}}{s}^{2}=-{\rm{d}}{Z}_{0}^{2}+{\rm{d}}{Z}_{1}^{2}+{\rm{d}}{Z}_{2}^{2}+{\rm{d}}{Z}_{3}^{3}+{\rm{d}}{Z}_{4}^{2}$$

and where *Λ* is the cosmological constant. The spherically symmetric coordinates (*T*, *R*, *θ*, *ϕ*) can be introduced by taking,

14 $$\begin{array}{rcl}{Z}_{0}={Z}_{0}^{{\prime} } & = & \sqrt{{a}^{2}-{R}_{0}^{2}}\sinh (T/a)\\ {Z}_{1}={Z}_{1}^{{\prime} } & = & \sqrt{{a}^{2}-{R}_{0}^{2}}\cosh (T/a)\\ {Z}_{2}={R}_{0}\cos ({\theta }_{0}),{Z}_{2}^{{\prime} } & = & {R}_{0}\cos ({\theta }_{0}^{{\prime} })\\ {Z}_{3}={R}_{0}\sin ({\theta }_{0} )\cos ({\phi }_{0} ),{Z}_{3}^{{\prime} } & = & {R}_{0}\sin ({\theta }_{0}^{{\prime} })\cos ({\phi }_{0}^{{\prime} })\\ {Z}_{4}={R}_{0}\sin ({\theta }_{0})\sin ({\phi }_{0}),{Z}_{4}^{{\prime} } & = & {R}_{0}\sin ({\theta }_{0}^{{\prime} })\sin ({\phi }_{0}^{{\prime} })\end{array}$$

where the unprimed embedding coordinates (*Z*_*i*_) represent one branch of the superposition, while the primed coordinates $({Z}_{i}^{{\prime} })$ represent the embedding coordinates of the other branch of the superposition. The two coordinate systems thus differ by rotations in the angular (*θ*) and azimuthal (*ϕ*) planes. In direct analogy to the prior example, one can write down similar dual representations of the metric and a test particle state as in Eqs. (10) and (11).

### Relativity of Spacetime Superpositions

#### Transition Amplitudes

Let us now apply the introduced assumptions and formalism to the problem of matter sourcing the metric that is in a superposition of diffeomorphically related states. We emphasise that the results presented here depend only on the linearity of quantum theory (insofar as symmetry transformations such as translations are described by unitary operators) and the invariance of physical laws under ordinary coordinate transformations. First, consider a source mass and the corresponding metric in a superposition of classically distinct configurations $\left|g(x)\right\rangle ,\left|g(y)\right\rangle$, described by the state

15 $$\left|\psi \right\rangle =\frac{1}{\sqrt{2}}\left(\left|g(x)\right\rangle +| g(y)\rangle\right)$$

(we generalize this to arbitrary superposition states in Methods: General Superposition States). It is helpful here to conceptualise the mass configuration as being quantum-controlled by an ancillary system that can be prepared and measured in appropriate states, in analogy with the standard approach in quantum measurement theory. That is, for each state of the control, there is an associated mass configuration with a classical manifold and gravitational field, relative to the other quantum DoFs. This is a key assumption that underpins numerous recent investigations in the area of spacetime superpositions, including analyses of gravitationally-induced entanglement proposals^22,23^, spacetime quantum reference frames^37,79^, and decoherence^25–27^.

According to Eq. (6), we can express the spacetime superposition state above as

16 $$\begin{array}{rcl}\left|\bar{\psi }\right\rangle & = & \frac{1}{\sqrt{2}}(\left|g(x)\right\rangle +{\widehat{T}}^{\dagger }(x,y)\left|g(x)\right\rangle )\\ & = & \frac{1}{\sqrt{2}}(\widehat{I}+{\widehat{T}}^{\dagger }(x,y))\left|g(x)\right\rangle .\end{array}$$

where by construction $\left|\bar{\psi }\right\rangle \equiv \left|\psi \right\rangle$ but we use this notation to distinguish the *representation* of the state from that in Eq. (15), though as emphasised, the two states are equivalent. We reiterate that this relationship follows from general relativity and the basic tenets of quantum theory, namely that symmetries can be represented with unitary operators. We also stress that in the case of translations, the spacetimes are related by a simple relabelling of, for example, the origin of coordinates, and not an active translation of the source mass through some pre-existing curved spacetime.

Let us now consider some additional DoF in the state $\left|{\phi }_{{\rm{DoF}}}\right\rangle$, and a joint initial state of the form,

17 $$\left|\psi \right\rangle =\frac{1}{\sqrt{2}}\left(\left|g(x)\right\rangle +\left|g(y)\right\rangle \right)\left|{\phi }_{{\rm{DoF}}}\right\rangle$$

where as discussed, the physical system represented by the state $\left|{\phi }_{{\rm{DoF}}}\right\rangle$ could be, for example, a quantum field or a particle in first-quantization, or both of these interacting with each other–we will consider all of these in the applications of our approach in the next sections and the Methods: Coupling a Quantum Field and Matter DoF to a Spacetime Superposition–Unruh-DeWitt Model. The only assumption behind Eq. (17) is that the state of the matter DoFs is uncorrelated with that of the source mass (and thus of the spacetime), but apart from this, it is arbitrary. Again, the assumption of an uncorrelated initial state is not necessary, and indeed, we will also consider correlated initial states in “Apparent Decoherence of Black Hole Superpositions”.

Let us consider the time evolution of the systems–including free dynamics as well as possible interactions between them–denoted by $\widehat{U}$. In general, the time evolution of the matter DoFs can depend on the state of the source mass, and so the operator $\widehat{U}$ can be represented as

18 $$\widehat{U}=\left|g(x)\right\rangle \langle g(x)| \otimes \widehat{U}(x)+| g(y)\rangle \left\langle g(y)\right|\otimes \widehat{U}(y),$$

where $\widehat{U}(x)$ and $\widehat{U}(y)$ individually govern the time-evolution of all the DoFs on the respective spacetime. The quantum-controlled evolution operator in Eq. (18) is consistent with the assumptions introduced in Preliminaries: Relativity of Superpositions, in the sense of a low-energy limit. We note that operators of this form are ubiquitous in, for example, quantum information theory–see for e.g. Refs. ^80–84^. Moreover, we note that Eq. (18) assumes that the free evolution of the individual metric states $\left|g(x)\right\rangle ,\left|g(y)\right\rangle$ is trivial. While this assumption holds in the examples considered, our formalism also directly applies to cases where the dynamics of the metric directly follow that of the source matter (as in the argument of Ref. ^44^ in Superposition of Geometries in the Gravitationally-Induced Entanglement Proposals).

Denoting $\left|{\Omega }_{{\rm{G}}}\right\rangle$ a final state of the gravitational DoFs, and by $\left|{\Omega }_{{\rm{DoF}}}\right\rangle$ the state of the matter DoFs, the general form of the probability amplitude reads

19 $$\begin{array}{l}\left\langle {\Omega }_{{\rm{DoF}}}\right|\langle {\Omega }_{{\rm{G}}}| \widehat{U}| \psi \rangle =\frac{1}{\sqrt{2}}\left(\left\langle {\Omega }_{{\rm{DoF}}}\right|\right.\langle {\Omega }_{{\rm{G}}}| \widehat{U}(x)| g(x)\rangle \\ \,\,+\left\langle {\Omega }_{{\rm{DoF}}}\right|\left.\langle {\Omega }_{{\rm{G}}}| \widehat{U}(y)| g(y)\rangle \right)\left|{\phi }_{{\rm{DoF}}}\right\rangle \end{array}$$

We wish to enact a transformation similar to (16) on the joint state of the metric and additional DoFs. Since changing the coordinates transforms the states of all involved systems, it acts both on the metric *g*(*y*) as well as on the additional DoFs *ϕ*_DoF_ in that branch of the superposition. That is, $\widehat{T}(x,y)={\widehat{T}}_{{\rm{G}}}(x,y)\otimes {\widehat{T}}_{{\rm{DoF}}}(x,y)\equiv {\widehat{T}}_{{\rm{G}}}\otimes {\widehat{T}}_{{\rm{DoF}}}$. Equation (19) thus becomes,

$$\begin{array}{l}\left\langle {\Omega }_{{\rm{DoF}}}\right|\langle {\Omega }_{{\rm{G}}}| \widehat{U}| \psi \rangle =\frac{1}{\sqrt{2}}\left\langle {\Omega }_{{\rm{DoF}}}\right|\langle {\Omega }_{{\rm{G}}}| \widehat{U}(x)| g(x)\rangle \left|{\phi }_{{\rm{DoF}}}\right\rangle \\ \,\,+\frac{1}{\sqrt{2}}\left\langle {\Omega }_{{\rm{DoF}}}\right|\langle {\Omega }_{{\rm{G}}}| \widehat{U}(y){\widehat{T}}^{\dagger }| g(x)\rangle \left|{\widehat{T}}_{{\rm{DoF}}}{\phi }_{{\rm{DoF}}}\right\rangle \end{array}$$

having dropped the explicit dependence on the coordinates *x*, *y* for brevity. Inserting the identity operator $\widehat{I}={\widehat{T}}^{\dagger }\widehat{T}$ in the second term gives,

20 $$\begin{array}{l}\left\langle {\Omega }_{{\rm{DoF}}}\right|\langle {\Omega }_{{\rm{G}}}| \widehat{U}| \psi \rangle =\frac{1}{\sqrt{2}}\left\langle {\Omega }_{{\rm{DoF}}}\right|\langle {\Omega }_{{\rm{G}}}| \widehat{U}(x)| g(x)\rangle \left|{\phi }_{{\rm{DoF}}}\right\rangle \\ \,\,+\frac{1}{\sqrt{2}}\left\langle {\widehat{T}}_{{\rm{DoF}}}{\Omega }_{{\rm{DoF}}}\right|\langle {\widehat{T}}_{{\rm{G}}}{\Omega }_{{\rm{G}}}| \widehat{T}\widehat{U}(y){\widehat{T}}^{\dagger }| g(x)\rangle \left|{\widehat{T}}_{{\rm{DoF}}}{\phi }_{{\rm{DoF}}}\right\rangle ,\end{array}$$

where the last line follows from $\left\langle {\widehat{T}}_{(\cdot )}{\Omega }_{(\cdot )}\right|=\left\langle {\Omega }_{(\cdot )}\right|{\widehat{T}}_{(\cdot )}^{\dagger }$. Importantly, as long as $\widehat{T}\equiv \widehat{T}(x,y)$ enacts a diffeomorphism, then

21 $$\widehat{T}\widehat{U}(y){\widehat{T}}^{\dagger }\equiv \widehat{U}(x)$$

which amounts to enacting a transformation of coordinates on the evolution operator on the second branch of the superposition, and thus we are left with the amplitude

22 $$\begin{array}{l}\left\langle {\Omega }_{{\rm{DoF}}}\right|\langle {\Omega }_{{\rm{G}}}| \widehat{U}| \psi \rangle =\frac{1}{\sqrt{2}}\left\langle {\Omega }_{{\rm{DoF}}}\right|\langle {\Omega }_{{\rm{G}}}| \widehat{U}(x)| g(x)\rangle \left|{\phi }_{{\rm{DoF}}}\right\rangle \\ \,\,+\frac{1}{\sqrt{2}}\left\langle {\widehat{T}}_{{\rm{DoF}}}{\Omega }_{{\rm{DoF}}}\right|\langle {\widehat{T}}_{{\rm{G}}}{\Omega }_{{\rm{G}}}| \widehat{U}(x)| g(x)\rangle \left|{\widehat{T}}_{{\rm{DoF}}}{\phi }_{{\rm{DoF}}}\right\rangle .\end{array}$$

Equations (19) and (22) describe the same scenario–they demonstrate that the same dynamics can be interpreted as taking place in spacetime that is in superposition of states $\left|g(x)\right\rangle ,\left|g(y)\right\rangle$ in Eq. (19), or in a single spacetime described by the state $\left|g(x)\right\rangle$, where now all the preparations and measurements are in superposition, as expressed in Eq. (22). We remark that this analysis follows analogously even if the gravitating source mass is in a spatial superposition of extended wavepackets. So long as one can relate the amplitudes of the superposition via an appropriate unitary transformation, the conclusions found here stand. In “Beyond Symmetries of Dynamics“, we show that this intuition also applies to scenarios where the relevant states are not related by a symmetry of the dynamics.

#### Full Interferometric Scenario

We now apply the above to a generic interferometric scenario, as this is most relevant to recent literature. In particular, consider the representation Eq. (19), where the source mass/spacetime is measured in the superposition basis, including the state $\left|{\Omega }_{{\rm{G}}}\right\rangle =(\left|g(x)\right\rangle +\left|g(y)\right\rangle )/\sqrt{2}$. For mutually orthogonal source mass/metric states, the final amplitude reads

23 $$\frac{1}{2}\langle {\Omega }_{{\rm{DoF}}}| (\widehat{U}(x)+\widehat{U}(y))| {\phi }_{{\rm{DoF}}}\rangle .$$

The interpretation of Eq. (23) is that matter DoFs prepared in the state $\left|{\phi }_{{\rm{DoF}}}\right\rangle$ evolve in a coherent superposition of “paths” associated with the different amplitudes of the spacetime superposition.

On the other hand, if we adopt the representation given by Eq. (22) the same amplitude equivalently reads

24 $$\begin{array}{l}\frac{1}{2}\langle {\Omega }_{{\rm{DoF}}}| \widehat{U}(x)| {\phi }_{{\rm{DoF}}}\rangle \\ +\frac{1}{2}\langle {\widehat{T}}_{{\rm{DoF}}}{\Omega }_{{\rm{DoF}}}| \widehat{U}(x)| {\widehat{T}}_{{\rm{DoF}}}{\phi }_{{\rm{DoF}}}\rangle ,\end{array}$$

where we used the fact that the two mass configurations and metrics are macroscopically distinct, meaning

$$\langle g(x)| {\widehat{T}}_{{\rm{G}}}^{\dagger }| g(x)\rangle =\langle g(x)| {\widehat{T}}_{{\rm{G}}}| g(x)\rangle =0$$

and again that $\widehat{T}\widehat{U}(y){\widehat{T}}^{\dagger }\equiv \widehat{U}(x)$.

The fact that Eqs. (23) and (24) are the same probability amplitude is a formal expression of the fact that a scenario involving quantum systems in a spacetime sourced by a spatial superposition of mass configurations is equivalent to a scenario where the particle follows different superposed trajectories in one spacetime and is measured as such. The physical interpretation of the probability amplitude is thus inherently ambiguous. The equivalence between the two scenarios can be interpreted as describing the same physical situation using two sets of coordinates that are related by a “superposition” of classical transformations^60^. The equivalence of amplitudes further implies that probabilities are identical in both scenarios.

This is a key point of this article and one that we believe is currently overlooked. “Quantum superpositions of spacetimes” can therefore be ambiguous, as in the example given here, as they can be re-expressed in terms of modified initial states and measurements of the remaining DoFs, but where the source mass, and thus the spacetime, can be treated as classical and remains fixed. This equivalence between representations is especially important given the recent interest in identifying quantum-gravitational effects that may arise from spatial superpositions of a source mass^10^.

**Fig. 3:**
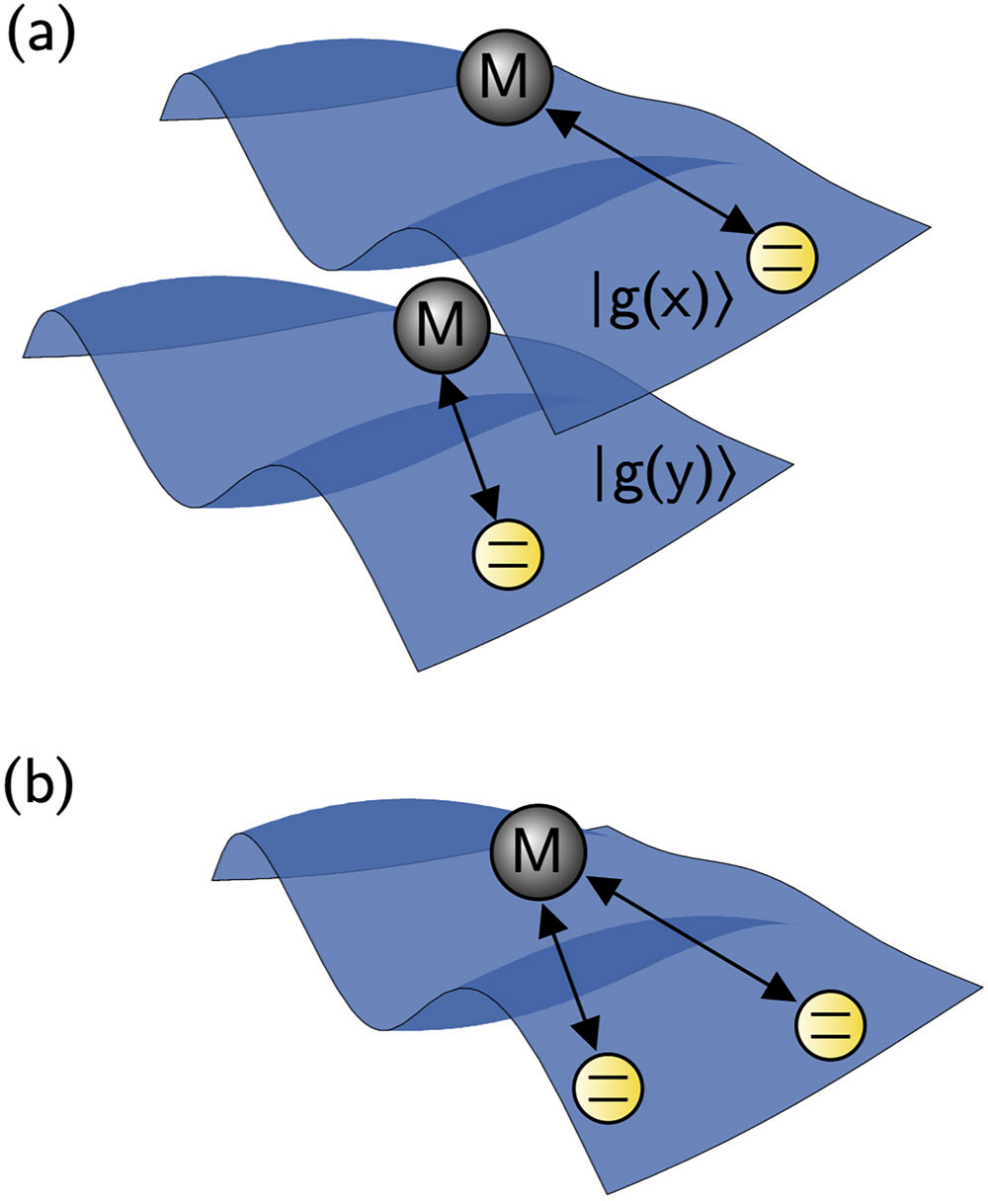
Equivalence of scenarios involving superpositions of spacetimes and matter degrees of freedom. Illustration of how the same scenario viewed from different coordinates acquires the interpretation of **a** superposition of spacetimes, **b** superposition of locations of matter DoFs (e.g., a test particle) with respect to one mass configuration. The curved “background” is illustrative only.

The crux of the argument is that only *relative configurations* between the interacting systems are physically relevant, such as a superposition of two distances between a pair of particles; a global (joint) coordinate transformation enacted on all degrees of freedom is irrelevant. Scenarios where quantum probes are situated on a classical background while the time-evolution occurs in a superposition of trajectories were recently considered in Refs. ^85–89^. For illustration, we have sketched schematically in Fig. 3 such a scenario where the superposed amplitudes differ by a translation.

Let us further take an approximation that the two amplitudes of the matter DoFs in Eq. (24) are orthogonal throughout the evolution, i.e. $\langle {\phi }_{{\rm{DoF}}}| {\widehat{T}}_{{\rm{DoF}}}{\phi }_{{\rm{DoF}}}\rangle =0$ and evolve only by a phase – which is also made in the gravitationally-induced entanglement proposals of Refs. ^22,23^. Here, this means that $\left|{\Omega }_{{\rm{DoF}}}^{+}\right\rangle :=(\left|{\phi }_{{\rm{DoF}}}\right\rangle +{e}^{i\varphi }\left|{\widehat{T}}_{{\rm{DoF}}}{\phi }_{{\rm{DoF}}}\right\rangle )/\sqrt{2}$ and defining analogously $\left|{\phi }_{\mathrm{DoF}}^{+}\right\rangle :=\left(\left|{\phi }_{\mathrm{DoF}}\right\rangle +\left|{\widehat{T}}_{\mathrm{DoF}}{\phi }_{\mathrm{DoF}}\right\rangle \right)/\sqrt{2}$ we obtain that Eq. (24) can be written as

25 $$\langle {\Omega }_{{\rm{DoF}}}^{+}| \widehat{U}(x)| {\phi }_{{\rm{DoF}}}^{+}\rangle .$$

This means that the scenario (a) in which the source mass is prepared and measured in superposition while the other DoFs evolve in the resulting superposition of metrics is physically equivalent and thus indistinguishable from the scenario (b) in which the other DoFs are prepared and measured in superposition while the source mass remains in some fixed state sourcing a classical metric.

We conclude this section by mentioning that the formalism presented here has a well-defined nonrelativistic limit both in terms of DoFs on the metric as well as source matter and metric. Indeed, our approach inherits the standard understanding of measurements in quantum field theory (e.g., spacetime smearing of position operators), which in the nonrelativistic limit is well-described by the position eigenstates utilized in “Preliminaries: Relativity of Superpositions“. In terms of source matter and metric, it can describe matter that sources a Newtonian potential and, through this potential, interacts with other massive particles. This is inherited in turn from the fact that the classical metric reduces to the Newtonian description in a low-energy, nonrelativistic limit. For source masses in quantum states, such a limit results in “superpositions of Newtonian potentials,” which have garnered just as much interest as their general relativistic counterparts (see, for example, Refs. ^49–51,53–58^). We now turn to specific scenarios of interest as applications of our results, starting from the above-mentioned superpositions of Newtonian potentials.

### Superposition of Geometries in the Gravitationally-Induced Entanglement Proposals

The previous analysis demonstrated how, in general, spacetime superpositions in which the respective amplitudes are related by a diffeomorphism can be re-expressed in terms of a single background spacetime with the states and measurements of the remaining DoFs being suitably adapted. This motivates us to revisit the conclusions one can draw concerning quantum features of gravity from such scenarios.

In this section, we apply our approach and construction to recent proposals by Bose et al. ^22^ and Marletto and Vedral^23^, which have attracted significant interest as presenting possibilities for witnessing the quantization of the gravitational field. These gravitationally-induced entanglement (GIE) setups suggest that observing entanglement between two spatially superposed source masses, interacting gravitationally via the Newtonian potential, is not equivalent to matter interferometry on a fixed background and would provide evidence of the quantum nature of gravity (for example, that entanglement is mediated via gravitons^90^). This was originally^22,23^ argued on the basis that local operations and classical communication cannot generate entanglement (LOCC), and thus, under the assumption that the interaction is mediated by a local DoF, any observed entanglement must have arisen because gravity acts as a quantum channel. We also draw attention to a recent interpretation of this argument by Christodolou and Rovelli^44^ who argue that such a setup represents a superposition of genuinely distinct spacetime metrics. This interpretation is the general relativistic generalization of related ones that interpret the GIE system as demonstrating a superposition of Newtonian gravitational fields (i.e., originally claimed in Ref. ^22^ and developed in later works e.g., Refs. ^50–52^).

**Fig. 4:**
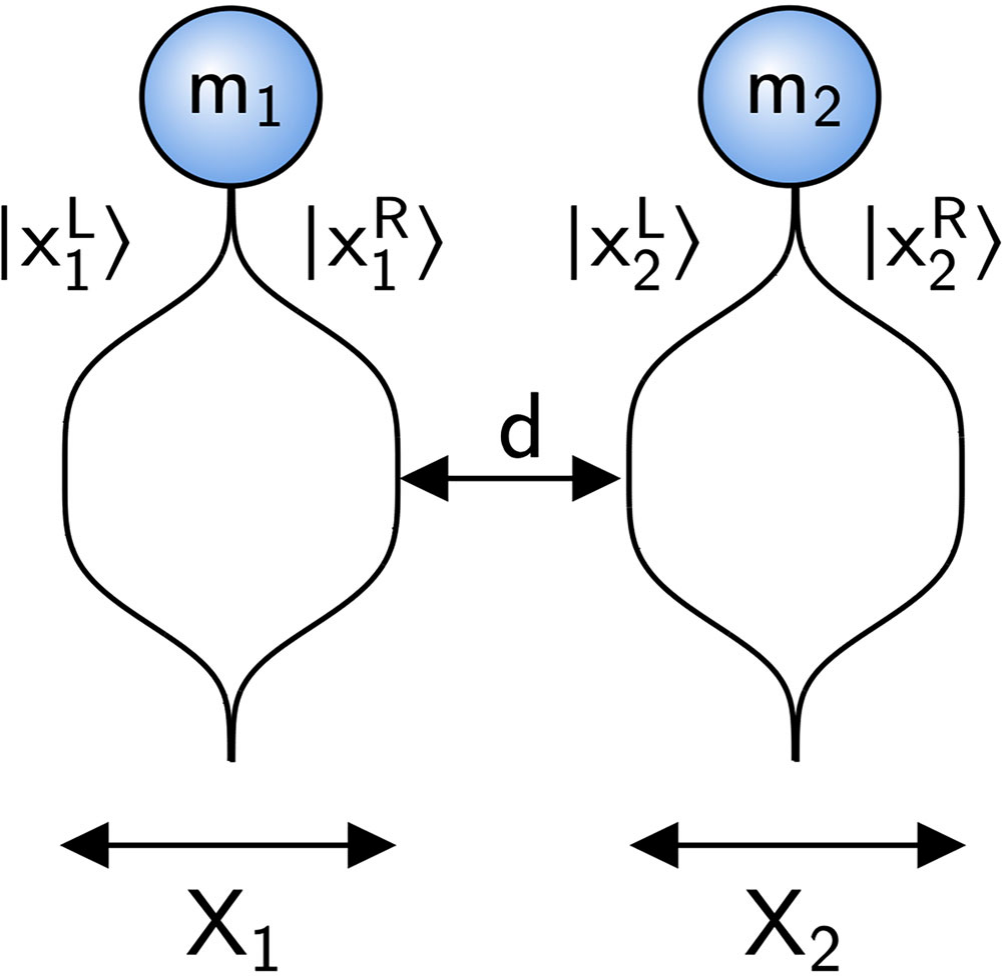
Schematic diagram of the GIE proposal. Each of the states $\left|{x}_{i}^{L,R}\right\rangle$ is assumed to be in a highly localised Gaussian wavepacket such that their overlap is zero.

The GIE proposal is depicted in Fig. 4. Two masses, *m*_1_ and *m*_2_, are each prepared and measured in spatial superposition of freely falling trajectories in the uniform gravitational field of Earth (for example, via Stern-Gerlach interferometry techniques). Thus initially, each particle is in a state of the form

$$\left|{\psi }_{\mathrm{GIE},i}\right\rangle =\frac{1}{\sqrt{2}}\left(\left|\psi ({x}_{i}^{L})\right\rangle +\left|\psi ({x}_{i}^{R})\right\rangle \right),$$

where *i* = 1, 2 labels the particles, while the labels *L*, *R* denote mutually orthogonal eigenstates of the *x*-component of the spin-1/2 particle, controlling the path that each particle takes. The states $\left|\psi ({x}_{i}^{A})\right\rangle$ are assumed to be highly localised trajectories, and thus we will henceforth denote them as position eigenstates $\left|{x}_{i}^{j}\right\rangle$. The initial joint state of the two particles is thus

26 $$\begin{array}{rcl}\left|{\psi }_{\mathrm{GIE}}\right\rangle & = & \left|{\psi }_{\mathrm{GIE},1}\right\rangle \otimes \left|{\psi }_{\mathrm{GIE,2}}\right\rangle \\ & = & \frac{1}{2}\left(\left|{x}_{1}^{L},{x}_{2}^{L}\right\rangle +\left|{x}_{1}^{L},{x}_{2}^{R}\right\rangle +\left|{x}_{1}^{R},{x}_{2}^{L}\right\rangle +\left|{x}_{1}^{R},{x}_{2}^{R}\right\rangle \right)\end{array}$$

Evolution under mutual gravitational interaction for a time *t* entangles the test masses by imparting distance-dependent phases to the components of the superposition. The simplifying assumption that the relative distance in one branch of the superposition state is much smaller than the others (in Fig. 4, $\left|{x}_{1}^{R},{x}_{2}^{L}\right\rangle$ with relative distance *d*) can be made, yielding the time-evolved state

27 $$\begin{array}{rcl}\left|{\psi }_{{\rm{GIE}}}\right\rangle & \to & \frac{1}{2}\left(\left|{x}_{1}^{L},{x}_{2}^{L}\right\rangle +\left|{x}_{1}^{L},{x}_{2}^{R}\right\rangle \right.\\ & & +\left.{e}^{i\phi }\left|{x}_{1}^{R},{x}_{2}^{L}\right\rangle +\left|{x}_{1}^{R},{x}_{2}^{R}\right\rangle \right)\end{array}$$

where *ϕ* = *Gm*_1_*m*_2_*t*/ℏ*d* is the phase shift induced by interaction via the Newtonian potential,

28 $$\widehat{U}=\int \,{\rm{d}}{x}_{1}{\rm{d}}{x}_{2}\,{e}^{\frac{iG{m}_{1}{m}_{2}t}{\hslash | {x}_{1}-{x}_{2}| }}\left|{x}_{1}\right\rangle \langle {x}_{1}| \otimes | {x}_{2}\rangle \left\langle {x}_{2}\right|$$

where the projectors $\left|{x}_{i}\right\rangle \left\langle {x}_{i}\right|$ act on the Hilbert space of the *i*th particle.

The argument of Ref. ^44^ is that immediately after the superpositions are created, the external metric $\left|g\right\rangle$ produced by both particles has not had time to change appreciably,

29 $$\left|{\psi }_{\mathrm{GIE}}^{(g)}\right\rangle =\frac{1}{2}\left|g\right\rangle \left(\left|{x}_{1}^{L}\right\rangle +\left|{x}_{1}^{R}\right\rangle \right)\left(\left|{x}_{2}^{L}\right\rangle +\left|{x}_{2}^{R}\right\rangle \right)=\frac{1}{2}\left|g\right\rangle \left(\left|{x}_{1}^{L},{x}_{2}^{L}\right\rangle +\left|{x}_{1}^{L},{x}_{2}^{R}\right\rangle +\left|{x}_{1}^{R},{x}_{2}^{L}\right\rangle +\left|{x}_{1}^{R},{x}_{2}^{R}\right\rangle \right)$$

We introduced the notation $\left|{\psi }_{{\rm{GIE}}}^{(g)}\right\rangle$ to highlight that this is the argument made in Ref. ^44^, distinct from Refs. ^22,23^ (for which we use the notation $\left|{\psi }_{{\rm{GIE}}}\right\rangle$). Once the gravitational disturbance has had time to propagate, the authors of Ref. ^44^ argue that each branch of the two-particle state will source, in general, a different metric depending on the relative distance between the particles in that branch:

30 $$\begin{array}{rcl}\left|{\psi }_{{\rm{GIE}}}^{(g)}\right\rangle & \to & \frac{1}{2}\left(\left|g({x}_{1}^{L},{x}_{2}^{L}),{x}_{1}^{L},{x}_{2}^{L}\right\rangle +\left|g({x}_{1}^{L},{x}_{2}^{R}),{x}_{1}^{L},{x}_{2}^{R}\right\rangle \right.\\ & + & \left.\left|g({x}_{1}^{R},{x}_{2}^{L}),{x}_{1}^{R},{x}_{2}^{L}\right\rangle +\left|g({x}_{1}^{R},{x}_{2}^{R}),{x}_{1}^{R},{x}_{2}^{R}\right\rangle \right)\end{array}$$

where $g({x}_{1}^{A},{x}_{2}^{B})$, *A*, *B* = *R*, *L* denote the respective two-body metrics sourced jointly by both particles at positions ${x}_{1}^{A},{x}_{2}^{B}$. They further remark that such a scenario no longer represents a semiclassical spacetime but a genuine superposition of metrics. This is true when referring to the metric generated by both particles.

However, despite the fact that Eq. (30) features four (in-principle) different states of the metric, the GIE proposal (nor its generalisation to a superposition of metrics in Ref. ^44^) does not depend on a superposition of geometries that is sourced by both particles. The results are derived from gravitational interaction *between* the two particles and thus the metric involved can be interpreted as sourced by, say, particle *i* = 1 (this choice being arbitrary), and the setup of Fig. 4 can be reinterpreted as a specific four-path interference experiment but on a fixed classical spacetime.

To see this explicitly (i.e. that GIE can be re-interpreted as though one of the particles sources a classical metric for the other), let us first consider the Hamiltonian of particle 2 with mass *m*_2_, with position ${\widehat{x}}_{2}$, in the background sourced by particle 1 with mass *m*_1_, with position ${\widehat{x}}_{1}$. Denoting the quantized relative distance between them as ${\widehat{x}}_{0}=| {\widehat{x}}_{2}-{\widehat{x}}_{1}|$, this is given by

31 $$\begin{array}{rcl}\widehat{H} & = & \sqrt{-{g}_{00}(\widehat{{x}_{0}})\left({m}_{2}^{2}{c}^{4}+{c}^{2}{g}_{ij}(\widehat{{x}_{0}}){\widehat{P}}^{i}{\widehat{P}}^{j}\right)}\\ & \to & \sqrt{-{g}_{00}({\widehat{x}}_{0}){m}_{2}^{2}{c}^{4}}\end{array}$$

where the notation ${g}_{00}(\widehat{{x}_{0}}),{g}_{ij}(\widehat{{x}_{0}})$ denotes that the metric components are functions of $\widehat{{x}_{0}}$, and $\widehat{{P}^{i}}$ for *i* = 1, 2, 3 are the components of the momentum operator (which we set to zero in the second line, since Refs. ^22,23,44^ assume fixed trajectories). Now, for the post-Newtonian background introduced earlier, Eq. (9), with components

32 $${g}_{00}=-\left(1+\frac{2\phi (\widehat{{x}_{0}})}{{c}^{2}}\right)$$

33 $${g}_{ij}={\delta }_{ij}\left(1-\frac{2\phi (\widehat{{x}_{0}})}{{c}^{2}}\right)$$

Eq. (31) simplifies to

34 $$\widehat{H}\simeq {m}_{2}{c}^{2}\left(1+\frac{\phi (\widehat{{x}_{0}})}{{c}^{2}}\right)$$

where the first term in the brackets will only contribute a global phase to the dynamics.

Before computing the dynamics, let us observe that the states $\left|{x}_{1}^{L}\right\rangle$, $\left|{x}_{1}^{R}\right\rangle$ are related by a unitary operator $\widehat{T}({X}_{1})\equiv {\widehat{T}}_{1}({X}_{1})\otimes {\widehat{T}}_{2}({X}_{1})$ (acting jointly on both particles) implementing a translation by the distance *X*_1_, namely (restoring also the metric state that depends on the location of both particles)

$$\begin{array}{l}\left|g({x}_{1}^{R},{x}_{2}^{L}),{x}_{1}^{R},{x}_{2}^{L}\right\rangle \\ \,={\widehat{T}}^{\dagger }({X}_{1})\left|g({x}_{1}^{L},{x}_{2}^{L}-{X}_{1}),{x}_{1}^{L},{x}_{2}^{L}-{X}_{1}\right\rangle \\ \left|{x}_{1}^{R},{x}_{2}^{R}\right\rangle \\ \,={\widehat{T}}^{\dagger }({X}_{1})\left|g({x}_{1}^{L},{x}_{2}^{R}-{X}_{1}),{x}_{1}^{L},{x}_{2}^{R}-{X}_{1}\right\rangle \end{array}$$

so that we can express the state of the particles and the metric in each branch from Eq. (30) as

35 $$\begin{array}{lll}\left|{\bar{\psi }}_{{\rm{GIE}}}^{(g)}\right\rangle = \frac{1}{2}\left(\left|g({x}_{1}^{L},{x}_{2}^{L}),{x}_{1}^{L},{x}_{2}^{L}\right\rangle +\left|g({x}_{1}^{L},{x}_{2}^{R}),{x}_{1}^{L},{x}_{2}^{R}\right\rangle \right.\\ \qquad\qquad\,+ \,\frac{\widehat{{T}^{\dagger }}}{2}\left|g({x}_{1}^{L},{x}_{2}^{L}-{X}_{1}),{x}_{1}^{L},{x}_{2}^{L}-{X}_{1}\right\rangle \\ \qquad\qquad\,+\,\frac{\widehat{{T}^{\dagger }}}{2}\left|g({x}_{1}^{L},{x}_{2}^{R}-{X}_{1}),{x}_{1}^{L},{x}_{2}^{R}-{X}_{1}\right\rangle .\end{array}$$

where $\left|{\bar{\psi }}_{{\rm{GIE}}}^{(g)}\right\rangle \equiv \left|{\psi }_{{\rm{GIE}}}^{(g)}\right\rangle$ as per our previous nomenclature for equivalent representations of the state, and we have suppressed the coordinate label of ${\widehat{T}}^{\dagger }$ for brevity. Let us stress that even though we have included the quantum state of the metric in the representation shown in Eq. (35), following Eq. (30), our final result–the equivalence of the probability amplitudes Eq. (40) and Eq. (41)–implies that this (and therefore the interpretation of a superposition of spacetime geometries) is not required to interpret the outcome of the experiment. In other words, no effects arising from a quantum superposition of geometries could be unambiguously verified through an interference experiment involving a state of the form Eq. (35). This is because the GIE setup only involves two particles mutually interacting via gravity, and as we demonstrate below, this means that the resulting phase shift (and probability amplitude) is reproduced in an equivalent representation in which one of the particles (here particle 1, though this choice is arbitrary) sources a classical gravitational field for the other, with suitably modified initial and final states. However, we utilize the metric notation here to clarify that all we have done thus far is suggestively rewritten the initial state, Eq. (30).

Now, in the GIE proposal, after some interval of time-evolution (under which part of the state acquires a relative phase), each particle is measured in a superposition by an uncorrelated measuring device. Following our previously introduced notation, we represent this measurement as

$$\left|{\Omega }_{{\rm{G}},{\rm{DoF}}}({x}_{1},{x}_{2})\right\rangle \equiv \left|\Omega ({x}_{1},{x}_{2})\right\rangle =\left|{\Omega }_{1}({x}_{1})\right\rangle \left|{\Omega }_{2}({x}_{2})\right\rangle$$

denoting the two particles and the metric associated with each of them, respectively. The metric component of the measurements state should be understood in a formal sense; we do not claim to provide a full description of measurements on gravitational DoFs as would be expected of a consistent theory of quantum gravity, but simply that in this low-energy regime, the metric is enslaved (i.e. perfectly correlated) to the position of the particle and thus measuring the particle’s position amounts to measuring the state of the metric sourced by the particle.

To proceed, let us compute the time-evolution generated by the Hamiltonian in Eq. (34), which for $\phi (\widehat{{x}_{0}})$ being the Newtonian potential, reduces to Eq. (28). The initial state, Eq. (35), becomes,

36 $$\begin{array}{lll}\widehat{U}\left|{\bar{\psi }}_{\mathrm{GIE}}^{(g)}\right\rangle = \frac{\widehat{U}}{2}\left(\left|g({x}_{1}^{L},{x}_{2}^{L}),{x}_{1}^{L},{x}_{2}^{L}\right\rangle +\left|g({x}_{1}^{L},{x}_{2}^{R}),{x}_{1}^{L},{x}_{2}^{R}\right\rangle \right)\\ \qquad\qquad\quad+\, \frac{\hat{U}{\hat{T}}^{\dagger }}{2}\left|g({x}_{1}^{L},{x}_{2}^{L}-{X}_{1}),{x}_{1}^{L},{x}_{2}^{L}-{X}_{1}\right\rangle \\ \qquad\qquad\quad+\, \frac{\hat{U}{\hat{T}}^{\dagger }}{2}\left|g({x}_{1}^{L},{x}_{2}^{R}-{X}_{1}),{x}_{1}^{L},{x}_{2}^{R}-{X}_{1}\right\rangle \end{array}$$

Next, using the general steps outlined in the framework introduced in Relativity of Spacetime Superpositions, we obtain the relevant probability amplitude by projecting the two-particle state onto $\left|\Omega \right\rangle \equiv \left|{\Omega }_{1}({x}_{1})\right\rangle \left|{\Omega }_{2}({x}_{2})\right\rangle \equiv \left|{\Omega }_{1}\right\rangle \left|{\Omega }_{2}\right\rangle$:

37 $$\begin{array}{l}\langle \Omega | \widehat{U}| {\bar{\psi }}_{\mathrm{GIE}}\rangle \\ =\frac{1}{2}\left\langle {\Omega }_{1}\right|\left\langle {\Omega }_{2}\right|\widehat{U}\left(\left|g({x}_{1}^{L},{x}_{2}^{L}),{x}_{1}^{L},{x}_{2}^{L}\right\rangle +\left|g({x}_{1}^{L},{x}_{2}^{R}),{x}_{1}^{L},{x}_{2}^{R}\right\rangle \right)\\ +\frac{1}{2}\left\langle \widehat{T}{\Omega }_{1}\right|\left\langle \widehat{T}{\Omega }_{2}\right|\widehat{T}\widehat{U}{\widehat{T}}^{\dagger }\left|g({x}_{1}^{L},{x}_{2}^{L}-{X}_{1}),{x}_{1}^{L},{x}_{2}^{L}-{X}_{1}\right\rangle \\ +\frac{1}{2}\left\langle \widehat{T}{\Omega }_{1}\right|\left\langle \widehat{T}{\Omega }_{2}\right|\widehat{T}\widehat{U}{\widehat{T}}^{\dagger }\left|g({x}_{1}^{L},{x}_{2}^{R}-{X}_{1}),{x}_{1}^{L},{x}_{2}^{R}-{X}_{1}\right\rangle \end{array}$$

where we have inserted the identity in the latter two amplitudes. We have denoted the action of the translation operator on the final state of the measurement as

$$\begin{array}{rcl}\left\langle \widehat{T}{\Omega }_{1}\right|\left\langle \widehat{T}{\Omega }_{2}\right| & \equiv & \left\langle {\Omega }_{1}({x}_{1}-{X}_{1})\right|\left\langle \Omega ({x}_{2}-{X}_{1})\right|\\ & = & \left\langle {\Omega }_{1}\right|\left\langle {\Omega }_{2}\right|{\widehat{T}}^{\dagger }\end{array}$$

in accordance with the nomenclature of Eqs. (8) and (20), where *x*_1_, *x*_2_ generically label the positions of particles 1, 2 (below we consider measurements performed on each particle in a superposition of paths). Note also that since the Newtonian potential is invariant under passive translations, $\widehat{T}\widehat{U}{\widehat{T}}^{\dagger }=\widehat{U}$, as the operator $\widehat{T}$ shifts the coordinates of both particles by the same distance. Finally, we emphasize that the effect of $\widehat{U}$ is to introduce a relative phase to the third term of Eq. (37), *ϕ* = *Gm*_1_*m*_2_*t*/ℏ*d*, which is identical to that obtained in the original formulation of the protocol under the assumptions stated previously^22,23^. With this understanding, we leave Eq. (37) with the evolution operators acting on the states in order to finalize our argument.

Consider now that the measurements are performed in a superposition of the paths, i.e.

38 $$\left|{\Omega }_{i}({x}_{i})\right\rangle =\frac{1}{\sqrt{2}}(\left|g({x}_{i}^{L}),{x}_{i}^{L}\right\rangle +\left|g({x}_{i}^{R}),{x}_{i}^{R}\right\rangle ).$$

The final probability amplitude in the original formulation i.e. Eq. (30), thus reads

39 $$\begin{array}{l}\langle \Omega | \widehat{U}| {\psi }_{{\rm{GIE}}}^{(g)}\rangle \\ =\frac{1}{4}\mathop{\sum }\limits_{A,B}\langle {x}_{1}^{A},{x}_{2}^{B},g({x}_{1}^{A},{x}_{2}^{B})| \widehat{U}| g({x}_{1}^{A},{x}_{2}^{B}),{x}_{1}^{A},{x}_{2}^{B}\rangle \end{array}$$

with the understanding that $\left|g({x}_{1}^{D})\right\rangle \left|g({x}_{2}^{{D}^{{\prime} }})\right\rangle \equiv \left|g({x}_{1}^{D},{x}_{2}^{{D}^{{\prime} }})\right\rangle$ represents the two-body metric associated with each branch of the superposition. At this point we can drop the metric notation $\left|g({x}_{1}^{D},{x}_{2}^{{D}^{{\prime} }})\right\rangle$ without loss of generality, giving an amplitude consistent with the original formulation, Eq. (26), which makes no reference to the metric:

40 $$\langle \Omega | \widehat{U}| {\psi }_{{\rm{GIE}}}\rangle =\frac{1}{4}\mathop{\sum }\limits_{A,B}\langle {x}_{1}^{A},{x}_{2}^{B}| \widehat{U}| {x}_{1}^{A},{x}_{2}^{B}\rangle$$

where the sum *A*, *B* = *L*, *R* runs over the four relevant amplitudes. We remark that this is Eq. (30) with the inclusion of time evolution and the final measurement; as discussed above, we retain the operatorial form of the evolution operators in Eq. (40) with the understanding that the third term accumulates a relative phase given in Eq. (27).

Now using our formalism, i.e., Eq. (35) leading to Eq. (37), the same amplitude reads

41 $$\langle {\Omega} | {\hat{U}}| {\bar{\psi }}_{{\rm{GIE}}}\rangle =\langle {\bar{x}}_{1},{\bar{x}}_{2}| {\hat{U}}| {\bar{x}}_{1},{\bar{x}}_{2}\rangle ,$$

where we suppressed the (*g*) superscript notation on the state, and where we have defined

42 $$\begin{array}{l}\left|{\bar{x}}_{1},{\bar{x}}_{2}\right\rangle :=\frac{1}{2}\left|{x}_{1}^{L}\right\rangle \otimes \left(\left|{x}_{2}^{L}\right\rangle +\left|{x}_{2}^{L}-{X}_{1}\right\rangle \right)\\ \,+\frac{1}{2}\left|{x}_{1}^{L}\right\rangle \otimes \left(\left|{x}_{2}^{R}\right\rangle +\left|{x}_{2}^{R}-{X}_{1}\right\rangle \right).\end{array}$$

Clearly we can write Eq. (41) as $\langle {\bar{x}}_{2}| \widehat{U}({\bar{x}}_{1})| {\bar{x}}_{2}\rangle$ where

43 $$\widehat{U}({\bar{x}}_{1})\equiv \langle {\bar{x}}_{1}| \widehat{U}| {\bar{x}}_{1}\rangle =\int \,{\rm{d}}{x}_{2}\,\exp \left(\frac{iG{m}_{1}{m}_{2}t}{\hslash | {x}_{1}^{L}-{\widehat{x}}_{2}| } \right)\left|{x}_{2}\right\rangle \left\langle {x}_{2}\right|$$

is the gravitational potential sourced by particle 1 positioned at ${x}_{1}^{L}$ and acting on the states of particle 2. Note that our nomenclature $\left|{\bar{x}}_{1}\right\rangle \equiv \left|{x}_{1}^{L}\right\rangle$ denotes the transformed final state in which particle 1 is measured, as per our formalism. Moreover, we remark that Eq. (41) may be interpreted as tacitly including metric sourced by particle *i* = 1, though clearly this can be interpreted without it, since the particle is interpreted as being on a classical trajectory.

The calculation above shows that GIE, originally explained as due to a quantum superposition of gravitational fields^22^, and subsequently as a superposition of two-body spacetime metrics^44^, can be explained in terms of a test mass prepared and measured in a superposition of four paths in the presence of a classical potential sourced by the other mass. Thus, there is an inherent ambiguity in the interpretation of such experiments as testing quantum features (i.e., the superposition principle) of gravitational degrees of freedom (i.e., semiclassical curved spacetime). Indeed, the latter interpretation is a variation of the celebrated COW (Collela-Overhauser-Werner) experiment^91^, in which neutrons in a Mach-Zehnder interferometer oriented within the Earth’s classical gravitational field experienced a gravitationally-induced phase shift dependent on the arm height. We stress that this ambiguity is present because in the considered scenario only relative distances between the particles play a role in determining the final probability amplitude, and not their absolute locations relative to, say, the laboratory reference frame.

Consequently, the interpretation that the initial state Eq. (29) evolves into Eq. (30) describing a superposition of semiclassical metrics sourced by the composite system comprised of both particles (the interpretation given by the authors of Ref. ^44^) while correct, is not required to predict the amplitude of interest in this proposal. Witnessing the effect of the superposition, in particular that described by the state given in Eq. (30) or, equivalently Eq. (35), would require a third test system residing in the spacetime jointly sourced by the two particles. In such a scenario, it is impossible to classically fix the positions of two of the systems via quantum coordinate transformations, as we show in the Methods: Breaking the Labelling Ambiguity with a Third System. It is not sufficient to treat the experimentalist in the laboratory (or more neutrally, a measuring device) as this third test system, unless one explicitly models the joint gravitational interaction between them (it) and the two particles. This would evidently constitute a modification to the usual description of the GIE proposal.

Instead, the result of the experiment can be adequately described in terms of a superposition of distances between masses, where the one designated as the source is fixed. Consequently, if we wanted to explicitly include a metric sourced by particle 1, it likewise could be interpreted as fixed. Our analysis emphasizes that any interpretation of a physical process should not be based on a particular mathematical representation of the states, which as highlighted above, is inherently ambiguous for any superposition of amplitudes differing by a symmetry of dynamics.

Removing the above ambiguity requires superpositions of non-diffeomorphic metrics. By definition such metrics are not related by any change of coordinates, since they effectively give rise to unique solutions to the Einstein field equations. It then follows that the prior analysis, in which various amplitudes in superposition could be related by coordinate transformations, is no longer applicable. In such cases, the superposition of geometries that arises is unambiguously quantum-gravitational, insofar as the resulting metrics associated with different amplitudes cannot be re-expressed as a single classical metric with suitably transformed dynamics and measurement bases for the remaining DoFs. Applied to the GIE proposals, this could involve atoms that are prepared in a superposition of energy eigenstates^92,93^, with each eigenstate generating a different curvature (such scenarios would likely be of conceptual, rather than of practical significance). Related examples that have been studied in the literature include a black hole^94,95^ or dark matter distribution^27^ in a superposition of masses, and an expanding universe in a superposition of expansion rates^88^.

Finally, let us remark that since the original GIE proposal can be interpreted within the Newtonian limit, the “metric” (or Newtonian potential) is fully characterized by the particle’s position. It is therefore in principle unnecessary to encode the metric DoFs in a separate Hilbert space, and one can work with states of the form Eq. (26). The introduction of a metric Hilbert space at the beginning of our analysis was to follow the conventions of Ref. ^44^, where it is explicitly argued that GIE realises a superposition of spacetime geometries.

### Apparent Decoherence of Black Hole Superpositions

In this section, we apply our approach to scenarios exploring the phenomenon of decoherence. We show how following a common approach may lead to different conclusions about the decoherence of a mass configuration, depending on how one defines the coordinate system to describe the configuration. Let us consider, for example, the case of a black hole at some position and its Hawking radiation, described by the state $\left|{\chi }_{{\rm{R}}}\right\rangle \equiv \left|{\chi }_{{\rm{R}}}({\bf{x}})\right\rangle$ with **x** being the spatial 3-vector of the black hole^25^. In this picture, a black hole in a superposition of different positions **x**, **y** i.e. sourcing the metrics *g*(**x**), *g*(**y**), becomes entangled with its own radiation

44 $$\left|\psi \right\rangle =\frac{1}{\sqrt{2}}\left(\left|g({\bf{x}}),{\chi }_{{\rm{R}}}({\bf{x}})\right\rangle +\left|g({\bf{y}}),{\chi }_{{\rm{R}}}({\bf{y}})\right\rangle \right.$$

Upon tracing out the radiation degrees of freedom from the state in Eq. (44), one finds using the basis $\{\left|g({\bf{x}})\right\rangle ,\left|g({\bf{y}})\right\rangle \}$

45 $${{\rm{Tr}}}_{{\rm{R}}}\left|\psi \right\rangle \left\langle \psi \right|=\frac{1}{2}\left(\begin{array}{cc}1 & \nu \\ {\nu }^{\star } & 1\end{array}\right)\to \frac{1}{2}\left(\begin{array}{cc}1 & 0\\ 0 & 1\end{array}\right),$$

where *ν* = 〈*χ*_R_(**x**)∣*χ*_R_(**y**)〉 is the overlap between the radiation states associated with the different positions of the black hole. For distinguishable states of the radiation, over time, the off-diagonal elements of the reduced density matrix will become suppressed, leaving a classical mixture of the two locations of the black hole. This has been argued to lead to fundamental decoherence^25,26,29^, due to the fact that a black hole cannot be isolated from its Hawking radiation and that the state of radiation depends on the location of the black hole.

However, the relevant question is, with respect to what physical system is the black-hole-radiation system superposed? Clearly, choosing coordinates whose origin is defined as the location of the black hole, often assumed as a natural choice, Eq. (44) by construction becomes a product state of the black hole and its radiation,

46 $$\left|\psi \right\rangle =\left|g({\bf{x}}),{\chi }_{{\rm{R}}}({\bf{x}})\right\rangle ,$$

from which one would not deduce decoherence. Based on our framework for the relativity of superpositions, the transformation between Eq. (44) and Eq. (46) involves or acts on all DoFs. The tacit assumption behind Eq. (45) is that in computing the overlap *ν*, one necessarily has access to an uncorrelated matter system ("measuring device(s)”), for example, a first-quantized particle that can interact with the radiation field *χ*_R_(**x**)^96,97^.

To see this, it is instructive to consider probability amplitudes that are obtained by coupling a measuring device whose position is correlated with the position of the black hole (the measuring device here is a proxy for arbitrary matter DoFs). Labelling the states of this device with its position **x** and some internal state *ϕ*_I_, i.e., $\left|{\phi }_{{\rm{I}}}({\bf{x}})\right\rangle$, let us consider the state of this device and the BH–radiation system

47 $$\begin{array}{l}\frac{1}{\sqrt{2}}\left|g({\bf{x}}),{\chi }_{{\rm{R}}}({\bf{x}})\right\rangle \left|{\phi }_{{\rm{I}}}({\bf{x}}+{\bf{d}})\right\rangle \\ \quad+\frac{1}{\sqrt{2}}\left|g({\bf{y}}),{\chi }_{{\rm{R}}}({\bf{y}})\right\rangle \left|{\phi }_{{\rm{I}}}({\bf{y}}+{\bf{d}})\right\rangle ,\end{array}$$

where the distance between the device and the black hole, ∣**d**∣, is identical in each branch of the superposition. Moreover, we assume that the two black hole positions are separated by a distance ∣**X**∣ and hence the states are related by the unitary transformation,

48 $$\left|g({\bf{y}}),{\chi }_{{\rm{R}}}({\bf{y}})\right\rangle \left|{\phi }_{{\rm{I}}}({\bf{y}}+{\bf{d}})\right\rangle ={\widehat{T}}^{\dagger }({\bf{X}})\left|g({\bf{x}}),{\chi }_{{\rm{R}}}({\bf{x}})\right\rangle \left|{\phi }_{{\rm{I}}}({\bf{x}}+{\bf{d}})\right\rangle .$$

Time evolution of the state is given by a unitary of the general form introduced in the earlier sections,

49 $$\widehat{U}=\left|g({\bf{x}})\right\rangle \left\langle g({\bf{x}})\right|\otimes \widehat{U}({\bf{x}})+\left|g({\bf{y}})\right\rangle \left\langle g({\bf{y}})\right|\otimes \widehat{U}({\bf{y}})$$

and where each $\widehat{U}({\bf{x}})$, $\widehat{U}({\bf{y}})$ describes an interaction between the measuring device and the radiation field for the *i*th position of the black hole. Note that here the metric in each amplitude remains stationary. Thus, applying Eq. (49) to evolve the initial state gives

50 $$\begin{array}{l}\frac{1}{\sqrt{2}}\widehat{U}({\bf{x}})\left|g({\bf{x}}),{\chi }_{{\rm{R}}}({\bf{x}})\right\rangle \left|{\phi }_{{\rm{I}}}({\bf{x}}+{\bf{d}})\right\rangle \\ \,+\frac{1}{\sqrt{2}}\widehat{U}({\bf{y}})\left|g({\bf{y}}),{\chi }_{{\rm{R}}}({\bf{y}})\right.\left|{\phi }_{{\rm{I}}}({\bf{y}}+{\bf{d}})\right\rangle .\end{array}$$

Using Eq. (48) and denoting $\left|{\phi }_{{\rm{I}}}^{{\prime} }({\bf{x}}+{\bf{d}})\right\rangle$ the state of the measuring device placed at distance ∣**d**∣ from the black hole after it has interacted with the radiation field, we find

51 $$\begin{array}{l}\frac{1}{\sqrt{2}}\left|g({\bf{x}}),{\chi }_{{\rm{R}}}({\bf{x}}),{\phi }_{{\rm{I}}}^{{\prime} }({\bf{x}}+{\bf{d}})\right\rangle \\\quad+\,\frac{1}{\sqrt{2}}{\widehat{T}}^{\dagger }({\bf{X}})\left|g({\bf{x}}),{\chi }_{{\rm{R}}}({\bf{x}}),{\phi }_{{\rm{I}}}^{{\prime} }({\bf{x}}+{\bf{d}})\right\rangle .\end{array}$$

This state factorizes for a scalar internal DoF such as rest mass-energy, i.e. since $\left|{\phi }_{{\rm{I}}}({\bf{x}}+{\bf{d}})\right\rangle =\left|{\phi }_{{\rm{I}}}({\bf{y}}+{\bf{d}})\right\rangle$ and $\left|{\phi }_{{\rm{I}}}^{{\prime} }({\bf{x}}+{\bf{d}})\right\rangle ={\widehat{T}}_{{\rm{I}}}^{\dagger }({\bf{X}})\left|{\phi }_{{\rm{I}}}^{{\prime} }({\bf{x}}+{\bf{d}})\right\rangle$ are unaffected by translations (nor will the final measurement basis $\left|{\Omega }_{{\rm{I}}}({\bf{x}}+{\bf{d}})\right\rangle$, see Eqs. (52) and (53)), so the total probability to find the internal DoF in some final state $\left|{\Omega }_{{\rm{I}}}({\bf{x}}+{\bf{d}})\right\rangle$ reads

52 $$\begin{array}{c}\frac{1}{2}{\left|g({\bf{x}}),{\chi }_{{\rm{R}}}({\bf{x}})\right\rangle +{\widehat{T}}^{\dagger }({\bf{X}})\left|g({\bf{x}}),{\chi }_{{\rm{R}}}({\bf{x}})\right\rangle }^{2}\\ \,\times {\left|\left\langle {\Omega }_{{\rm{I}}}({\bf{x}}+{\bf{d}})| {\phi }_{{\rm{I}}}^{{\prime} }({\bf{x}}+{\bf{d}})\right\rangle \right|}^{2}\end{array}$$

Assuming the orthogonality for position states of the black hole implies that

$$\langle {\chi }_{{\rm{R}}}({\bf{x}}),g({\bf{x}})| \widehat{T}({\bf{X}})| g({\bf{x}}),{\chi }_{{\rm{R}}}({\bf{x}})\rangle =0$$

which further means that Eq. (52) simplifies to

53 $$\begin{array}{l}{\left|\langle {\chi }_{{\rm{R}}}({\bf{x}}),g({\bf{x}})| g({\bf{x}}),{\chi }_{{\rm{R}}}({\bf{x}})\rangle \right|}^{2}{\left|\langle {\Omega }_{{\rm{I}}}({\bf{x}}+{\bf{d}})| {\phi }_{{\rm{I}}}^{{\prime} }({\bf{x}}+{\bf{d}})\rangle \right|}^{2}\\ \quad={\left|\langle {\Omega }_{{\rm{I}}}({\bf{x}}+{\bf{d}})| {\phi }_{{\rm{I}}}^{{\prime} }({\bf{x}}+{\bf{d}})\rangle \right|}^{2}\end{array}$$

which is exactly the same probability as we get in the case when the positions of the measuring device, the black hole, and the radiation field are fixed i.e. the black hole has a “classical” relative distance with respect to the measuring device (note that for a coordinate-dependent internal DoF like spin, Eq. (52) will no longer factorize–in such a case, the measurement can be interpreted as occurring in a correlated manner analogous to Eq. (93). This does not affect the conclusion of our analysis, Eq. (53)). If we consider all other DoFs in the universe are likewise correlated, all observables will be consistent with a classical position of the black hole and will not allow us to conclude that there is any decoherence. Our analysis may be viewed as a consequence of the principle of background independence discussed in Preliminaries: Relativity of Superpositions. Specifically, the states $\left|g((x),{\chi }_{{\rm{R}}}({\bf{x}})\right\rangle \left|{\phi }_{{\rm{I}}}({\bf{x}}+{\bf{d}})\right\rangle$ and $\left|g((y),{\chi }_{{\rm{R}}}({\bf{y}})\right\rangle \left|{\phi }_{{\rm{I}}}({\bf{y}}+{\bf{d}})\right\rangle$ belong to the same equivalence class of physical spacetimes plus matter fields (and non-relativistic DoFs, here the internal state of the detector) under diffeomorphisms. Superpositions thereof are physically indistinguishable from the respective states individually. In the language of Ref. ^42^, these are superpositions of models living on the same orbit, which are deemed “false” superpositions.

We note that the assumption that the radiation states $\left|{\chi }_{i}\right\rangle$ are related by a translation is consistent with the treatment in Ref. ^25^ where the density matrix of the radiation in the momentum basis is taken to be

54 $$\left|{\chi }_{{\rm{R}}}({\bf{x}})\right\rangle \left\langle {\chi }_{{\rm{R}}}({\bf{y}})\right|=\int \,{{\rm{d}}}^{3}{\bf{k}}\frac{p(| {\bf{k}}| )}{4{\pi }^{2}| {\bf{k}}| }{e}^{-i{\bf{k}}\cdot {\bf{x}}}\left|{\bf{k}}\right\rangle \left\langle {\bf{k}}\right|{e}^{i{\bf{k}}\cdot {\bf{y}}},$$

where *p*(∣**k**∣) is the probability of emitting a particle with momentum ∣**k**∣ and **x**, **y** are the spatial 3-vectors associated with the coordinate systems *x*, *y* respectively. (Eq. (54), taken from Ref. ^25^, assumes plane waves for the radiation in the region asymptotically far from the black hole.) This, in particular, implies that 〈*χ*_R_(**y**)∣*χ*_R_(**x**)〉 is only a function of ∣**x** − **y**∣, see Ref. ^25^ for an explicit expression.

The alternative scenario is to consider a measuring device whose position is not correlated with the position of the black hole, for example described by the state

55 $$\frac{1}{\sqrt{2}}\left(\left|g({\bf{x}}),{\chi }_{{\rm{R}}}({\bf{x}})\right\rangle +\left|g({\bf{y}}),{\chi }_{{\rm{R}}}({\bf{y}})\right\rangle \right)\left|{\phi }_{{\rm{I}}}({\bf{z}})\right\rangle$$

where the position of the device **z** is uncorrelated with the position of the black hole, and so the device’s relative distance to the black hole is now different in each amplitude, i.e. **z** = **x** + **d**_1_ = **y** + **d**_2_ with **d**_1_ ≠ **d**_2_. In this case, Eq. (51) reads

$$\begin{array}{l}\frac{1}{\sqrt{2}}\left|g({\bf{x}}),{\chi }_{{\rm{R}}}({\bf{x}})\right\rangle \left|{\phi }_{{\rm{I}}}^{{\prime} }({\bf{x}}+{{\bf{d}}}_{1})\right\rangle \\\quad+\,\frac{1}{\sqrt{2}}{\widehat{T}}^{\dagger }(X)\left|g({\bf{x}}),{\chi }_{{\rm{R}}}({\bf{x}})\right\rangle \left|{\phi }_{{\rm{I}}}^{{\prime} }({\bf{x}}+{{\bf{d}}}_{2})\right\rangle ,\end{array}$$

and the state of the measuring device in general gets correlated with the black-hole-radiation system, which in turn leads to suppression of the off-diagonal terms of the black-hole radiations system given by the overlap between the states of the measurement device $\langle {\phi }_{{\rm{I}}}^{{\prime} }\left.({\bf{x}}+{{\bf{d}}}_{1})\right)| {\phi }_{{\rm{I}}}^{{\prime} }({\bf{x}}+{{\bf{d}}}_{2})\rangle$. Here, we can conclude that there will be decoherence over time as information is exchanged between the black hole and the measuring device through the radiation at the location of the device. Note, however, that for any translation-invariant interaction, the information is only encoded in the distances **d**_*i*_ between the device and the black hole. When this distance is equal in both branches, i.e., Eq. (51), decoherence does not arise since $\langle {\phi }_{{\rm{I}}}^{{\prime} }({\bf{d}})| {\phi }_{{\rm{I}}}^{{\prime} }({\bf{d}})\rangle =1$.

The conclusion is therefore that one should rather speak about the decoherence of the relative distance between a black hole and other systems that can obtain information about the black-hole-radiation system. In this sense, decoherence of black holes due to Hawking radiation does not need to be interpreted as fundamental, since in the absence of any external DoFs, the position of the black hole is not even unambiguously defined. This is related to the point made by Unruh and referred to as “false loss of coherence” ^98^. There, the observation is that decoherence is often inferred by virtue of the coupling between a system and its environment; however, as long as any changes to the system are made adiabatically, when the superposed amplitudes are brought together, the state of the environment will also be brought together and will not lead to decoherence. Here, however, we do consider that the radiation keeps being emitted over time. The two scenarios are then: is there any external system that can read out the difference as in Eq. (55), or is all external matter also correlated with the black hole as in Eq. (47)? In the latter case, the superposition state can be regarded as only due to a choice of coordinates.

### Coherence of Spacetime Superpositions

We have argued in the preceding section that the presence of matter that can encode information about the black hole is crucial to consider, even if the DoFs of the radiation from the black hole are included in the analysis. We now apply our framework to analyze quantitatively how much spacetime superpositions decohere due to the presence of such matter DoFs.

Let us consider, for concreteness, the case where a probe particle resides outside a black hole in a superposition of relative distances from the horizon. For simplicity, we model the particle as a qubit with scalar-valued internal states $\left|{\phi }_{{\rm{I}}}\right\rangle \equiv \left|{\phi }_{{\rm{I}}}({\bf{z}})\right\rangle \in \{\left|0\right\rangle ,\left|1\right\rangle \}$ (where **z** is the 3-position of the probe), whose evolution depends on the metric and the probe’s position through interaction with a quantum field $\widehat{\phi }$, whose initial state is denoted by $\left|{\phi }_{{\rm{F}}}\right\rangle$. To summarise, the initial state of the black hole, probe internal DoF, and quantum field is given by

$$\left|\psi \right\rangle =\frac{1}{\sqrt{2}}(\left|g({\bf{x}})\right\rangle +\left|g({\bf{y}})\right\rangle )\left|{\phi }_{{\rm{I}}}\right\rangle \left|{\phi }_{{\rm{F}}}\right\rangle$$

where in keeping with the nomenclature of Superposition of Geometries in the Gravitationally-Induced Entanglement Proposals, **x**, **y** denote the spatial 3-vector of the black hole (though we could equally replace these with generic coordinate systems following the notation of Relativity of Spacetime Superpositions; see also Methods: Unruh-DeWitt Model). Like Eq. (55), the position of the probe particle is uncorrelated with the black hole’s position. The evolution takes the form

$$\widehat{U}\left|\psi \right\rangle =\frac{1}{\sqrt{2}}(\widehat{U}({\bf{x}})\left|g({\bf{x}})\right\rangle +\widehat{U}({\bf{y}})\left|g({\bf{y}})\right\rangle )\left|{\phi }_{{\rm{I}}}\right\rangle \left|{\phi }_{{\rm{F}}}\right\rangle .$$

Note that, in addition to the uncorrelated probe state, we assumed that the field $\left|{\phi }_{{\rm{F}}}\right\rangle$ factorizes from the black-hole position. This is a particular choice inspired by a recent investigation of the (2+1)-dimensional Banados-Zanelli-Teitelboim black hole, where the ground state of the field was taken to be the anti-de Sitter vacuum state satisfying this assumption^94^. However, the method employed here is not constrained by this choice; i.e., one could as well assume the field state to be correlated with the black hole.

To compute the decoherence of the black hole, we can consider an interferometric setup in which the black hole (or the control system correlated with the black hole) undergoes a Mach-Zehnder-type trajectory with a controllable phase *φ* on one of the arms^30^ and is then measured in a superposition basis (in line with the description provided in Results: Full Interferometric Scenario); for clarity we compute probability amplitudes for the following final superposition state $\left|{\Omega }_{{\rm{G}}}\right\rangle =(\left|g({\bf{x}})\right\rangle +{e}^{-i\varphi }\left|g({\bf{y}})\right\rangle )/\sqrt{2}$, giving the following conditional state of the internal and field DoFs,

56 $$\left|{\psi }_{{\rm{IF}}}\right\rangle \equiv \langle {\Omega }_{{\rm{G}}}| \widehat{U}| \psi \rangle =\frac{1}{2}(\widehat{U}({\bf{x}})+{e}^{i\varphi }\widehat{U}({\bf{y}}))\left|{\phi }_{{\rm{I}}}\right\rangle \left|{\phi }_{{\rm{F}}}\right\rangle$$

with density matrix ${\rho }_{{\rm{IF}}}\equiv \left|{\psi }_{{\rm{IF}}}\right\rangle \left\langle {\psi }_{{\rm{IF}}}\right|$

57 $$\begin{array}{rcl}{\rho }_{{\rm{IF}}} & = & \frac{1}{4}\widehat{U}({\bf{x}}){\rho }_{{\rm{IF}}}(0){\widehat{U}}^{\dagger }({\bf{x}})+\frac{1}{4}\widehat{U}({\bf{y}}){\rho }_{{\rm{IF}}}(0){\widehat{U}}^{\dagger }({\bf{y}})\\ & & \,+\frac{{e}^{-i\varphi }}{4}\widehat{U}({\bf{x}}){\rho }_{{\rm{IF}}}(0){\widehat{U}}^{\dagger }({\bf{y}})+{\rm{h.c}}\end{array}$$

where the Hermitian conjugate applies to the last term in Eq. (57) and *ρ*_IF_(0) denotes the initial state of the internal and field DoFs. Tracing out the internal and field DoFs leaves

58 $${{\rm{Tr}}}_{{\rm{IF}}}\left[\langle {\Omega }_{{\rm{G}}}| \rho (t)| {\Omega }_{{\rm{G}}}\rangle \right]=\frac{1}{2}(1+v\cos (\varphi)),$$

where *ρ*(*t*) is the time-evolved state of the full system. We denote $v=| \langle {\phi }_{{\rm{F}}}| \langle {\phi }_{{\rm{I}}}| \widehat{U}{({\bf{x}})}^{\dagger }\widehat{U}({\bf{y}})| {\phi }_{{\rm{I}}}\rangle | {\phi }_{{\rm{F}}}\rangle |$ as the interferometric visibility and *φ* is a relative phase, i.e., *v* is the contrast between the maxima and minima of the interference fringes obtained as *φ* is varied. If *v* = 0, the black hole state has fully decohered, whereas if *v* = 1, coherence is maximally retained. Importantly, *v* is explicitly given by the distinguishability of the states of the probe and field DoFs after the interaction. In general, the visibility will take the form (see Methods: Unruh-DeWitt Model)

59 $$v=1+{L}_{{\bf{x}}{\bf{y}}}-\left({P}_{{\bf{x}}}+{P}_{{\bf{y}}}\right)/2.$$

Here, *L*_**xy**_ is a contribution originating as an interference term between the superposed amplitudes, while *P*_**x**_ and *P*_**y**_ are contributions from the individual amplitudes themselves. In Methods “Unruh-DeWitt Model”, we give an explicit form for these terms in a particular example of an interaction known as the Unruh-DeWitt model^99–101^, which can be taken to approximate the light-matter interaction^102^. In general, *P*_1,2_ > *L*_12_ and thus *v* < 1: some amount of coherence is lost. However, in the regime of weak coupling between the qubit, field, and spacetime, the decrease in coherence will be very small, ~*O*(*λ*^2^) where *λ* ≪ 1. This example is fully consistent with Unruh’s observation concerning rapid or strong interactions leading to decoherence, while weak coupling or adiabatic evolution leads to false loss of coherence^103–106^. Moreover, it aligns with the intuition that decoherence results from the transfer of information from the system to the environment^98^, where a weak coupling allows for some of the coherence to be retained.

Of course, as more probe particles are introduced into the system, the decrease in visibility becomes more significant. The visibility for a system of *N* particles coupled to the black hole is given by

60 $$v=1+\frac{1}{2}\mathop{\sum }\limits_{i\ne j}^{N}{L}_{ij}-\frac{1}{2}\mathop{\sum }\limits_{i}^{N}{P}_{i}.$$

where the sums run over all the amplitudes of the superposition. Here, *L*_*ij*_ is the interference term between the *i*th and *j*th components of the superposition (i.e., analogous to *L*_**xy**_ above), while *P*_*i*_ are the “local” contributions from the *i*th amplitude of the superposition (i.e., analogous to *P*_**x**_, *P*_**y**_ above). The factor of (1/2) in front of the first summation is to avoid double-counting the *L*_*ij*_ = *L*_*ji*_ terms. Since *P*_*i*_ > *L*_*ij*_ for all (*i*, *j*) in general, Eq. (60) can be interpreted that as a macroscopic amount of matter is considered, even weakly interacting with the superposition, the decoherence (loss of visibility) becomes very strong.

## Discussion

In this article, we have introduced the notion of “relativity of spacetime superpositions” for quantum superpositions of source-mass configurations whose amplitudes differ by a coordinate transformation. Such superposition states have garnered significant interest due to their direct relevance in low-energy tests of quantum gravity, and for understanding emergent quantum-gravitational phenomena from the “bottom-up” (i.e., investigations of quantum-gravitational effects that do not rely upon a formal theory of quantum gravity). Our framework only depends upon the basic tenets of linearity of quantum theory and the invariance of dynamics under coordinate transformations. We have drawn attention to the fact that the choice of labeling configurations of physics systems, even when it involves the notion of superposition or entanglement and the systems are sources of a gravitational field, is not fundamental but rather merely conventional. So long as the involved states are related by a coordinate transformation, their associated semiclassical metrics by definition also map to one another. Consequently, any such scenario can be mapped to a scenario in which the metric is fixed and classical, while the states and measurements of remaining DoFs are appropriately modified. While such a resulting scenario in general may not appear natural, our point is that it gives rise to the possibility of making equivalent predictions. Our approach is complementary to recent work in the field of QRFs, as it does not require considering additional degrees of freedom of the reference frames, as the linearity of quantum theory and the representation of symmetries via unitary operators are sufficient for the results.

The relativity of spacetime superpositions has significant implications for both conceptual and practical proposals for witnessing quantum-gravitational effects arising from so-called quantum superpositions of geometries. The main point emerging from our framework is that quantum superpositions of gravitational sources whose states are related by a coordinate transformation (which includes all spatial and even temporal superpositions) are not *unambiguously* quantum-gravitational, insofar as they can be described in terms of preparation and measurement of quantum systems on a classical background. This is particularly important in ongoing discussions and proposals for deriving and observing phenomena not describable within the current paradigms of quantum mechanics and classical general relativity.

A further application of our results is in the discussion on decoherence. Our examples show that it is often tacitly assumed but not explicitly stated that the conclusions require considering that specific measurements can be performed on environmental degrees of freedom. Here, we have argued that such measurements are not only required to see decoherence, but only through such measurements can superpositions of mass configurations acquire physical meaning. Without them, and assuming complete isolation from all externally coupled systems, superpositions of spatial states of spacetime are operationally indistinguishable from dynamics occurring on a single classical background. This highlights the importance of including all interactions with matter DoFs in the analysis of the dynamics of spacetime superpositions, such as those considered in Ref. ^25^ (there, it is the tacitly assumed probe that couples to the position of the gravitational source mass). However, as we have demonstrated, the inclusion of such matter does not make the superposition more fundamentally “quantum-gravitational,” for it is again always possible to re-express the spacetime as a single, fixed background and the matter in a corresponding superposition of configurations.

Before concluding this article, we note very recent work by Kabel et al. ^42^ that looks at a similar problem to the one we address here, namely the question of what it means to identify points across spacetimes in superposition. In the spirit of Refs. ^107–110^, Kabel et al. adopt the language of models (*φ*), which are points in the state space of a physical theory (*Φ*). In the case of general relativity, a given model *φ* = (*M*, *g*, *ψ*_DoF_) consists of a manifold *M*, Lorentzian metric *g*, and in general, additional DoFs *ψ*_DoF_. The symmetry group here is the diffeomorphism group Diff(*M*), and the space of models is partitioned into orbits (i.e., the collection of states that result from applying all sets of possible symmetry transformations on a given initial state, traditionally an equivalence class) of this group. For general relativity, this means that one orbit contains all diffeomorphically equivalent spacetimes, and models in the same orbit are deemed physically indistinguishable. Removing redundancy in this prescription is analogous to gauge fixing in gauge theories, and in the spirit of fixing coordinates already discussed in “Preliminaries: Relativity of Superpositions”-“Preliminaries: Examples”, is merely a “representational convention.” This choice of a representational convention (which fixes a way to choose a representative model from each orbit) corresponds to the choice of a QRF relative to which the state of a system is described, while the associated counterpart relation prescribes how to compare two models $\varphi ,{\varphi }^{{\prime} }$.

In brief: a unique representational convention for a given orbit can be chosen via the injective map

61 $$\sigma :[\Phi ]\to \Phi$$

62 $$[\varphi ]\mapsto \sigma ([\varphi ])$$

where [*Φ*] denotes the space of equivalence classes [*φ*] with respect to the equivalence relation “ ~ ” which is defined by $\varphi \sim {\varphi }^{{\prime} }$ iff there exists a *g* such that ${\varphi }^{{\prime} }={\varphi }^{g}$ (*φ*^*g*^ denotes the result of acting on *φ* ∈ *Φ* with *g* ∈ *G* where *G* is the symmetry group of interest (here, *G* = Diff(*M*))). The counterpart relation,

63 $${{\rm{Counter}}}_{\sigma }(\varphi ,{\varphi }^{{\prime} })={g}_{\sigma }{(\varphi^{{\prime}} )}^{-1}\circ {g}_{\sigma }(\varphi )$$

is an element of *G* and defines how to move from *φ* to ${\varphi }^{{\prime} }$ within *Φ*, see Fig. 3 of Ref. ^42^.

The relevant insight of Ref. ^42^ is that the choice of a representational convention (or QRF) changes how one prescribes the identification of points across the different branches of a superposition of spacetimes. Thus, the concept of localization (i.e., the designation of which system is in superposition) is frame-dependent, generalizing this phenomenon from the nonrelativistic case^36^. The authors introduce a set of fields ${\chi }_{i},{\widetilde{\chi }}_{i}$ that define bijective maps from *M* to ${{\mathbb{R}}}^{4}$ (i.e., they are assumed to be inhomogeneous in such a way that identification of points across branches of a superposition is unique), with the subscript *i* = 1, 2 denoting the branch of the superposition on which these fields reside (for a twofold superposition). The choice of the reference field (i.e., either $\chi ,\widetilde{\chi }$) fixes a representational convention–with their respective values used to label points of the spacetime manifold. These $\chi ,\widetilde{\chi }$ fields then define a counterpart relation that defines a natural way of comparing points across the different branches of the superposition: two points in different branches are considered “identical” whenever they share the same values of $\chi ,\widetilde{\chi }$ respectively. Crucially, the choice of either $\chi ,\widetilde{\chi }$ as the representational convention gives rise to two inequivalent comparison maps used to compare points across the different branches of the superposition.

The spirit of our framework is closely aligned with that of Ref. ^42^. In particular, both approaches are grounded in the principle of background independence, where it is natural to “mod out” those spacetimes that are diffeomorphic. In Ref. ^42^, the choice of the reference system (the fields $\chi ,\widetilde{\chi }$) determines a unique prescription for identifying points across the branches, as well as the localization of other involved systems; in our framework, the arbitrary choice of coordinates used to label a quantum state of the spacetime leads to different interpretations concerning what systems (including the spacetime itself) are “in superposition.” While we focus on a different question than Kabel et al., it should be possible to reframe our results in their language, and vice versa. We also emphasize that unlike Ref. ^42^, and the QRF literature more broadly, our framework is not built around associating a reference frame with a specific quantum system. Rather, we focus on the problem of how the arbitrary choice of coordinates used to label states–which in general relativity is merely conventional–extends to conventions where the involved states (here, superpositions of semiclassical spacetimes) are not related by a classical transformation. Nevertheless, while the typical approach is to associate a quantum state with a concrete physical system (like a particle) that acts as the QRF (when seen relative to another QRF), this can be understood in an abstract sense, too. In Ref. ^42^, Kabel et al. consider the possibility of using “abstract or idealised coordinate fields,” which is in the spirit of our treatment of coordinate systems, which we simply use as labels (but do not associate with them a quantum state).

As alluded to above, the symmetry between representations of scenarios involving superpositions of spatial configurations is broken once one considers superpositions of metrics that cannot be related by a diffeomorphism. An example of such a scenario is a source in a superposition of masses, as the associated states of the gravitational field represent unique solutions to Einstein’s field equations and thus *are* physically distinguishable. This point motivates looking for extensions to the current GIE proposals, namely experimental schemes involving superpositions of non-diffeomorphic metrics, and the possibility of witnessing quantum-gravitational effects induced thereby. Finally, let us remark about the scenario considered in “Apparent Decoherence of Black Hole Superpositions”. As we have emphasized throughout, two spacetime configurations related by a diffeomorphism are physically equivalent, and thus, no observable—if it is to respect diffeomorphism invariance—can distinguish between the scenario in which the metric is in superposition, compared with the case where all other systems are in superposition on a fixed background. The two scenarios considered in “Apparent Decoherence of Black Hole Superpositions” (i.e., when the detector is correlated with the spacetime, and when it factorizes from it) are two distinct scenarios, and therefore give two distinct outcomes. This does not mean that the choice of initial state is able to break the ambiguity concerning what constitutes a “genuine” superposition of spacetimes, but simply that the dynamics of the detector will depend on this choice.

## Methods

### Coupling a Quantum Field and Matter DoF to a Spacetime Superposition

In this section, we apply the relativity of spacetime superpositions to the specific interaction between a quantum field and a first-quantized particle with the background sourced by a mass in superposition of configurations. For simplicity, we assume that the particle is pointlike and thereby couples to the field along a semiclassical trajectory.

Consider, as usual, the initial state of the mass configuration, field, and the particle (i.e., matter) to be

64 $$\left|\psi \right\rangle =\frac{1}{\sqrt{2}}(\left|g(x)\right\rangle +\left|g(y)\right\rangle )\left|{\phi }_{{\rm{M}}}\right\rangle \left|{\phi }_{{\rm{F}}}\right\rangle .$$

where *x*, *y* are arbitrary coordinates describing the configurations *g*(*x*), *g*(*y*). We assume that the particle can be held static in each amplitude of the superposition without necessarily obtaining which-way information about its position relative to the mass. The total Hilbert space of the relevant systems is given by ${\mathcal{H}}={{\mathcal{H}}}_{{\rm{S}}}\otimes {{\mathcal{H}}}_{{\rm{M}}}\otimes {{\mathcal{H}}}_{{\rm{F}}}$ where ${{\mathcal{H}}}_{{\rm{S}}}$, ${{\mathcal{H}}}_{{\rm{M}}}$, and ${{\mathcal{H}}}_{{\rm{F}}}$ are respectively associated with the spacetime, matter, and field DoFs.

Let us consider the following general form of the interaction Hamiltonian, ${\widehat{H}}_{{\rm{I}}}$, which couples all degrees of freedom:

65 $${\widehat{H}}_{{\rm{I}}}=\left|g(x)\right\rangle \langle g(x)| \otimes \widehat{H}(x;{x}_{{\rm{M}}})+| g(y)\rangle \left\langle g(y)\right|\otimes \widehat{H}(y;{y}_{{\rm{M}}})$$

where *x*_M_, *y*_M_ denotes the worldline along which the particle interacts with the field, which in general can be considered to be the same or different for the two mass configurations; however in Eq. (64) we assume the former for simplicity. In sum, the Hamiltonian (65) couples the field and matter at location *x*_M_, *y*_M_ for the mass configuration $\left|g(x)\right\rangle ,\left|g(y)\right\rangle$. For distinguishable mass configurations, the assigned states $\left|g(x)\right\rangle$, $\left|g(y)\right\rangle$ are mutually orthogonal, and the time evolution operator in the interaction picture between the initial time *t*_*i*_ and final time *t*_*f*_ is given by

66 $$\widehat{U}=\exp \left(-i{\int }_{{t}_{i}}^{{t}_{f}}{\rm{d}}t\frac{{\rm{d}}\tau }{{\rm{d}}t}{\widehat{H}}_{{\rm{I}}}\right),$$

where *τ* is the proper time of the particle on the worldline associated with *x*_M_, *y*_M_. $\widehat{U}$ can be written generally as

67 $$\widehat{U}=\left|g(x)\right\rangle \langle g(x)| \otimes \widehat{U}(x;{x}_{{\rm{M}}})+| g(y)\rangle \left\langle g(y)\right|\otimes \widehat{U}(y;{y}_{{\rm{M}}}),$$

where $\widehat{U}(z;{z}_{{\rm{M}}})$ is the unitary operator describing the interaction for a given $\left|g(z)\right\rangle$ mass configuration (serving here as a “control”), with *z* = *x*, *y*.

After the interaction and upon measuring the control in the state $\left|{\Omega }_{{\rm{G}}}\right\rangle =(\left|g(x)\right\rangle +\left|g(y)\right\rangle )/\sqrt{2}$, the final state of the remaining DoFs (here, the field and the internal state of the particle) is given by

68 $$\langle {\Omega }_{{\rm{G}}}| \widehat{U}| \psi \rangle =\frac{1}{2}(\widehat{U}(x)+\widehat{U}(y))\left|{\phi }_{{\rm{M}}}\right\rangle \left|{\phi }_{{\rm{F}}}\right\rangle ,$$

where we have suppressed the dependence on *x*_M_, *y*_M_ for brevity. Note that Eq. (68) is a conditional state of the field and a particle. Considering now that the position of the probe particle is the same in either amplitude, *x*_M_ = *y*_M_ = *x*_0_ (as assigned by the agent who describes the mass configurations as *g*(*x*), *g*(*y*)), the scenario can be interpreted as the particle at *x* interacting with a field quantized on a background in a superposition of geometries *g*(*x*), *g*(*y*).

An alternative interpretation is to consider the scenario from the “quantum coordinates” associated with the mass, in which there is a single, fixed background where the particle is interacting with the field in a superposition of different locations. As argued in the main text, the coordinates assigned to the joint state of the control states with the field $\left|g(x)\right\rangle \left|{\phi }_{{\rm{F}}}\right\rangle$, $\left|g(y)\right\rangle \left|{\phi }_{{\rm{F}}}\right\rangle$ can be related using an operator $\widehat{T}(x,y)$, Eqs. (5) and (6), that connects the coordinates parametrizing the two relative configurations of the mass with respect to the matter DoFs. Assuming for simplicity that *g*(*x*), *g*(*y*) are related by a translation *X*, so that $\widehat{T}(x,y)=\widehat{T(X)}$, we can write

69 $$\begin{array}{l}\left|g(y)\right\rangle \left|{\phi }_{{\rm{M}}}\right\rangle \left|{\phi }_{{\rm{F}}}\right\rangle \\ ={\widehat{T}}^{\dagger }(X)\left|g(x)\right\rangle \left|{\widehat{T}}_{{\rm{M}}}{\phi }_{{\rm{M}}}\right\rangle \left|{\widehat{T}}_{{\rm{F}}}{\phi }_{{\rm{F}}}\right\rangle \\ ={\widehat{T}}_{{\rm{G}}}^{\dagger }(X)\left|g(x)\right\rangle \otimes {\widehat{T}}_{{\rm{M}}}^{\dagger }(X)\left|{\widehat{T}}_{{\rm{M}}}{\phi }_{{\rm{M}}}\right\rangle \otimes {\widehat{T}}_{{\rm{F}}}^{\dagger }(X)\left|{\widehat{T}}_{{\rm{F}}}{\phi }_{{\rm{F}}}\right\rangle \end{array}$$

where recall the notation ${\widehat{T}}_{i}^{\dagger }\left|{\widehat{T}}_{i}{\phi }_{i}\right\rangle \equiv \left|{\phi }_{i}\right\rangle$ for the ancillary DoF *ϕ*_*i*_. The $\widehat{T}(X)$ operator enacts a translation on the entire system. For clarity, we have explicitly written above that it consists here of ${\widehat{T}}_{{\rm{G}}}(X)$, which acts on the source mass and thus also on the associated metric, and ${\widehat{T}}_{{\rm{F}}}(X)$ which acts on the coordinates of the field operator. That is,

$$\widehat{\phi }(x-X)={\widehat{T}}_{{\rm{F}}}(X)\widehat{\phi }(x){\widehat{T}}_{{\rm{F}}}^{\dagger }(X).$$

On the level of the unitary, the transformation thus gives

$$\widehat{U}(x;{y}_{{\rm{M}}}-X)=\widehat{T}(X)\widehat{U}(y;{y}_{{\rm{M}}}){\widehat{T}}^{\dagger }(X).$$

The state of the system is now

$$\widehat{U}\left|\bar{\psi }\right\rangle =\frac{1}{\sqrt{2}}\widehat{U}(\widehat{I}+{\widehat{T}}^{\dagger }(X))\left|g(x)\right\rangle \left|{\phi }_{{\rm{M}}}\right\rangle \left|{\phi }_{{\rm{F}}}\right\rangle ,$$

where again, we have used the label $\left|\bar{\psi }\right\rangle \equiv \left|\psi \right\rangle$ to clarify that though this is an equivalent physical situation to that described by Eq. (68), the change of the representation of the state from $\left|\psi \right\rangle$ to $\left|\bar{\psi }\right\rangle$ gives this situation a different interpretation. After projecting onto the control, again in the superposition state $\left|{\bar{\Omega }}_{{\rm{G}}}\right\rangle =(\widehat{I}+{\widehat{T}}^{\dagger }(X))\left|g(x)\right\rangle /\sqrt{2}$, we obtain

70 $$\begin{array}{l}\langle {\bar{\Omega }}_{{\rm{G}}}| \widehat{U}| \bar{\psi }\rangle \\ =\frac{1}{2}\langle g(x)| (\widehat{I}+\widehat{T}(X))\widehat{U}(\widehat{I}+{\widehat{T}}^{\dagger }(X))| g(x)\rangle \left|{\widehat{T}}_{{\rm{M}}}{\phi }_{{\rm{M}}}\right\rangle \left|{\widehat{T}}_{{\rm{F}}}{\phi }_{{\rm{F}}}\right\rangle .\end{array}$$

Since

$$\begin{array}{l}\widehat{T}(x,y)\widehat{U}(y;{y}_{{\rm{M}}})\left|g(y)\right\rangle \left\langle g(y)\right|{\widehat{T}}^{\dagger }(x,y)\\ \,\,=\widehat{U}(x;{y}_{{\rm{M}}}-X)\left|g(x)\right\rangle \left\langle g(x)\right|\end{array}$$

and recognising that

$$\langle g(x)| \widehat{T}(X)\widehat{U}| g(x)\rangle =\langle g(x)| \widehat{U}{\widehat{T}}^{\dagger }(X)| g(x)\rangle =0$$

due to the assumption of mutual orthogonality of the states $\left|g(x,y)\right\rangle$ this simplifies to

71 $$\begin{array}{l}\langle {\bar{\Omega }}_{{\rm{G}}}| \widehat{U}| \bar{\psi }\rangle \\\quad=\frac{1}{2}\langle g(x)| \left(\widehat{U}(x;{x}_{{\rm{M}}})+\widehat{U}(x;{y}_{{\rm{M}}}-X)\right.| g(x)\rangle \left|{\widehat{T}}_{{\rm{M}}}{\phi }_{{\rm{M}}}\right\rangle \left|{\widehat{T}}_{{\rm{F}}}{\phi }_{{\rm{F}}}\right\rangle .\end{array}$$

This amplitude can be interpreted as the field and the qubit interacting on a single metric. Following the special case considered after Eq. (68), when *x*_M_ = *y*_M_ ≡ *x*_0_, we now have that the particle interacts with the field in a superposition of different locations, *x*_0_ and *x*_0_ − *X*. Since by construction $\langle {\bar{\Omega }}_{{\rm{G}}}| \widehat{U}| \bar{\psi }\rangle \equiv \langle {\Omega }_{{\rm{G}}}| \widehat{U}| \psi \rangle$, physical observables–which could be transition amplitudes between some states of the field and matter–are invariant between the two representations. This further highlights the equivalence of the two interpretations of this scenario: as a superposition of different states of the source mass with the particle interacting with a field at *x* or as a single state of the mass configuration with the matter (particle) in a spatial superposition.

### General Superposition States

In this section, we generalise the results shown in “Discussion” to generic superposition states of the metric:

72 $$\left|\psi \right\rangle =\beta \left|g({x}_{0})\right\rangle +\mathop{\sum }\limits_{i\ne 0}{\alpha }_{i}\left|g({x}_{i})\right\rangle ,$$

where ∣*β*∣^2^ + ∑_*i*_∣*α*_*i*_∣^2^ = 1, and *x*_*i*_ parametrise the coordinate system of the *i*th spacetime amplitude and 〈*g*(*x*_*i*_)∣*g*(*x*_*j*_)〉 = *δ*_*ij*_. Again, we assume that the spacetimes are related by diffeomorphisms, allowing for the states $\left|g({x}_{i})\right\rangle$ to be related to a background metric state $\left|g({x}_{0})\right\rangle$, via a unitary $\left|g({x}_{i})\right\rangle ={\widehat{T}}^{\dagger }({x}_{i},{x}_{0})\left|g({x}_{0})\right\rangle$. As before, we describe some quantum DoFs in the state $\left|\phi \right\rangle \equiv \left|{\phi }_{{\rm{DoF}}}\right\rangle$ interacting with the spacetime superposition through the time-evolution governed by $\widehat{U}={\sum }_{i\ne 0}\widehat{U}({x}_{i})\left|g({x}_{i})\right\rangle \left\langle g({x}_{i})\right|$, before performing a general measurement of the system in the state $\left|\Omega \right\rangle$ (describing all DoFs including the metric):

$$\begin{array}{l}\langle \Omega | \widehat{U}| \psi \rangle =\beta \langle \Omega | \widehat{U}({x}_{0})| g({x}_{0})\rangle \left|\phi \right\rangle \\ \,\,+\mathop{\sum }\limits_{i\ne 0}{\alpha }_{i}\langle \Omega | \widehat{U}({x}_{i})| g({x}_{i})\rangle \left|\phi \right\rangle .\end{array}$$

Following the same procedure as before, we find that the dynamics can be expressed in such a way as to occur on a single spacetime metric $\left|g({x}_{0})\right\rangle$ while the other DoFs are in a superposition of states and the measurements are found via the transformations $\widehat{T}$:

73 $$\begin{array}{l}\langle \Omega | \widehat{U}| \psi \rangle =\beta \langle \Omega | \widehat{U}({x}_{0})| g({x}_{0})\rangle \left|\phi \right\rangle \\ \,\,+\mathop{\sum }\limits_{i\ne 0}{\alpha }_{i}\langle {\widehat{T}}_{i}\Omega | {\widehat{T}}_{i}\widehat{U}({x}_{i}){\widehat{T}}_{i}^{\dagger }| g({x}_{i})\rangle \left|{\widehat{T}}_{i}\phi \right\rangle .\end{array}$$

where ${\widehat{T}}_{i}\widehat{U}({x}_{i}){\widehat{T}}_{i}^{\dagger }=\widehat{U}({x}_{0})$ and the notation ${\widehat{T}}_{i}\equiv \widehat{T}({x}_{i},{x}_{0})$ is understood. Following the main text, let us also look at the scenario where the metric undergoes a complete evolution through an “interferometric” setup (i.e., where the state of the control or spacetime is measured in an appropriate basis where interference can be witnessed). For an initial state of the spacetime and matter DoFs (the latter in the state $\left|\phi \right\rangle \equiv \left|{\phi }_{{\rm{DoF}}}\right\rangle$) given by

74 $$\left|\psi \right\rangle =\beta \left|g({x}_{0})\right\rangle \left|\phi \right\rangle +\mathop{\sum }\limits_{i\ne 0}{\alpha }_{i}\left|g({x}_{i})\right\rangle \left|\phi \right\rangle$$

one can always express the time-evolved state as some modified dynamics on a classical background metric *g*(*x*_0_):

75 $$\begin{array}{l}\widehat{U}\left|\bar{\psi }\right\rangle =\widehat{U}\left(\beta +\mathop{\sum }\limits_{i\ne 0}{\alpha }_{i}{\widehat{T}}^{\dagger }\right)\left|g({x}_{0})\right\rangle \left|\phi \right\rangle \\ \,\,=\widehat{U}({x}_{0})\beta \left|g({x}_{0})\right\rangle \left|\phi \right\rangle +\mathop{\sum }\limits_{i\ne 0}{\alpha }_{i}\widehat{U}({x}_{i}){\widehat{T}}_{i}^{\dagger }\left|g({x}_{0})\right\rangle \left|{\widehat{T}}_{i}\phi \right\rangle \end{array}$$

where in the second line we adopt the nomenclature of Eq. (8), and we have used the notation $\left|\psi \right\rangle \equiv \left|\bar{\psi }\right\rangle$ as usual to distinguish the different representations of the same physical situation. Projecting the spacetime (i.e., the control) state in the superposition basis, already expressed using the unitary $\widehat{T}$ i.e.

76 $$\left|{\bar{\Omega }}_{{\rm{G}}}\right\rangle =\beta \left|g({x}_{0})\right\rangle +\mathop{\sum }\limits_{i\ne 0}{\alpha }_{i}{\widehat{T}}_{i}^{\dagger }\left|g({x}_{0})\right\rangle$$

yields the final amplitude

77 $$\begin{array}{l}\langle {\bar{\Omega }}_{{\rm{G}}}| \widehat{U}| \bar{\psi }\rangle \\\quad=\langle g({x}_{0})| \left(| \beta {| }^{2}\widehat{U}+\mathop{\sum }\limits_{i,j\ne 0}{\alpha }_{i}^{\star }{\alpha }_{j}{\widehat{T}}_{i}\widehat{U}{\widehat{T}}_{j}^{\dagger }\right)| g({x}_{0})\rangle \left|{\widehat{T}}_{i}\phi \right\rangle ,\\\quad\equiv | \beta {| }^{2}\widehat{U}({x}_{0}) \left| \phi \right\rangle+\mathop{\sum }\limits_{i\ne 0}| {\alpha }_{i}{| }^{2}\widehat{U}({x}_{i}) \left|{\widehat{T}}_{i}\phi \right\rangle .\end{array}$$

This is, of course, the same result one would obtain if preparing and measuring the spacetime in a superposition of states that are mutually related to each other via some diffeomorphism encoded within $\widehat{T}$. The diffeomorphism invariance of the two scenarios shows that one can always map such a superposition of geometries–something that is believed to be beyond the formalism of quantum theory on a fixed background–onto a single spacetime metric.

### Unruh-DeWitt Model

In this section, we apply a specific interaction model to calculate the coherence of a generic spacetime superposition coupled to a quantum field and matter DoF modelled as a qubit. We utilize the Unruh-DeWitt model (UdW) used widely in relativistic quantum information and analogue gravity settings.

For simplicity, let us consider a superposition of two spacetime states $\left|g(x)\right\rangle$, $\left|g(y)\right\rangle$, such that the initial (product) state of all DoFs is given by $\left|\psi \right\rangle =(\left|g(x)\right\rangle +\left|g(y)\right\rangle )\left|0\right\rangle \left|\phi \right\rangle /\sqrt{2}$ where $\left|0\right\rangle$ is the ground state of the UdW detector and $\left|\phi \right\rangle$, the state of the field, is assumed to be the same for either coordinate parametrization *g*(*x*), *g*(*y*) (though one could in-principle consider detector or field states entangled with the metric). Note also that we use *x*, *y* to parametrize two coordinate systems, in keeping with the notation of Preliminaries: Relativity of Superpositions, though one could also perform this analysis by treating *x* → **x**, *y* → **y** as describing the position of a source mass, as considered in Superposition of Geometries in the Gravitationally-Induced Entanglement Proposals. We work in the interaction picture, with the interaction between all DoFs described by the Hamiltonian,

78 $$\begin{array}{rcl}{\widehat{H}}_{{\rm{I}}} & = & \lambda \widehat{\sigma }(\tau )\eta (\tau )\left(\left|g(x)\right\rangle \right.\left\langle g(x)\right|\otimes \widehat{\phi }(x)\\ & & \,+\left|g(y)\right\rangle \left\langle g(y)\right|\otimes \widehat{\phi }\left.(y)\right).\end{array}$$

Here, *λ* ≪ 1 is a weak coupling constant and

79 $$\widehat{\sigma }=\left|1\right\rangle \left\langle 0\right|{e}^{iE\tau }+{\rm{H.c.}}$$

is the *SU*(2) ladder operator between the internal states of the qubit $\{\left|0\right\rangle ,\left|1\right\rangle \}$ with energy gap *E*. The quantities *x*, *y* are the coordinates of the point where the qubit interacts with the field, and may in principle be different on different branches of the superposition, and *η*(*τ*) is a “switching function” parametrized by the proper time of the qubit (its role is defining when the interaction is switched on–it is often chosen to be a Gaussian or a function with a compact support). Note that we have chosen to factor $\widehat{\sigma }(\tau )$, *η*(*τ*) from the control, which in turn may imply different coordinate times associated with the same *τ* on the two manifolds. One can also describe the scenario from the perspective of a common coordinate time (e.g. associated with sufficiently far away clock) in which case the individual proper times may be different depending on the position of the qubit relative to the source mass.

The time-evolution operator can be expanded in the Dyson series

$$\widehat{U}=\widehat{{\mathbb{I}}}+{\widehat{U}}^{(1)}+{\widehat{U}}^{(2)}+O({\lambda }^{3}),$$

up to second-order in the weak coupling constant *λ*, where

80 $${\widehat{U}}^{(1)}=-i{\int }_{-\infty }^{+\infty }{\rm{d}}\tau \,{\widehat{H}}_{{\rm{I}}}(\tau ),$$

81 $${\widehat{U}}^{(2)}=-{\int }_{-\infty }^{+\infty }{\rm{d}}\tau {\int }_{-\infty }^{\tau }{\rm{d}}{\tau }^{{\prime} }\,{\widehat{H}}_{{\rm{I}}}(\tau ){\widehat{H}}_{{\rm{I}}}({\tau }^{{\prime} }),$$

are the first- and second-order terms. Note that the integration bounds of Eq. (81) enforce time-ordering of the time-dependent Hamiltonians ${\widehat{H}}_{{\rm{I}}}(\tau ),{\widehat{H}}_{{\rm{I}}}({\tau }^{{\prime} })$. The time-evolution takes the usual form,

$$\widehat{U}=\left|g(x)\right\rangle \langle g(x)| \otimes \widehat{U}(x)+| g(y)\rangle \left\langle g(y)\right|\otimes \widehat{U}(y),$$

where

$$\begin{array}{rcl}\widehat{U}(x) & = & \widehat{I}+{\widehat{U}}_{1}(x)+{\widehat{U}}_{2}(x)+O({\lambda }^{3}),\\ \widehat{U}(y) & = & \widehat{I}+{\widehat{U}}_{1}(y)+{\widehat{U}}_{2}(y)+O({\lambda }^{3}),\end{array}$$

describe the time-evolutions that depend on the spacetime states $\left|g(x)\right\rangle$ and $\left|g(y)\right\rangle$ respectively, while ${\widehat{U}}_{1}(x,y),{\widehat{U}}_{2}(x,y)$ are the “local” unitaries evaluated with respect to the coordinates *x*, *y*, at first- and second-order in perturbation theory respectively. The time-evolved state is

82 $$\begin{array}{rcl}\widehat{\rho } & = & \widehat{U}\left|\psi \right\rangle \left\langle \psi \right|{\widehat{U}}^{\dagger }\\ & = & \frac{1}{2}\mathop{\sum }\limits_{z,{z}^{{\prime} }}\hat{U}(z)\left|g(z)\right\rangle \left\langle g({z}^{{\prime} })\right|\otimes {\rho }_{\mathrm{DF}}{\widehat{U}}^{\dagger }({z}^{{\prime} })\end{array}$$

where the sum is over the relevant amplitudes $z,{z}^{{\prime} }=x,y$ and ${\widehat{\rho }}_{{\rm{DF}}}$ is the density matrix of the detector-field subsystem. The conditional state of the detector-field subsystem, after projecting onto the spacetime state $\left|{\Omega }_{{\rm{G}}}\right\rangle =(\left|g(x)\right\rangle +{e}^{i\varphi }\left|g(y)\right\rangle )/\sqrt{2}$ where *φ* is some controllable phase, is,

83 $$\begin{array}{rcl}{\rho }_{\mathrm{DF}}(t) & = & \frac{1}{2}\hat{U}(x){\rho }_{\mathrm{DF}}{\widehat{U}}^{\dagger }(x)+\frac{1}{2}{\hat{U}}(y){\rho }_{{\mathrm{DF}}}{\hat{U}}^{\dagger }(y)\\ && \, +\frac{{e}^{-i\varphi }}{2}{\hat{U}}(x){\rho }_{{\mathrm{DF}}}{\hat{U}}^{\dagger }(y)+{\mathrm{h}}.{\mathrm{c}}\end{array}$$

where h.c denotes the Hermitian conjugate applied to the last term. Tracing over the remaining DoFs gives,

84 $$\begin{array}{rcl}{{\rm{Tr}}}_{{\rm{DF}}}\left[\langle {\Omega }_{{\rm{G}}}| \rho (t)| {\Omega }_{{\rm{G}}}\rangle \right] & = & \frac{1}{4}\left(\langle {\widehat{U}}^{\dagger }(x)\widehat{U}(x)\rangle +\langle {\widehat{U}}^{\dagger }(y)\widehat{U}(y)\rangle \right.\\ & & \left.+{e}^{-i\varphi }\langle {\widehat{U}}^{\dagger }(y)\widehat{U}(x)\rangle +{\rm{h.c}}\right)\end{array}$$

where the expectation values are taken with respect to the detector-field state $\left|0\right\rangle \left|\phi \right\rangle$ (note also that $\widehat{U}(x),\widehat{U}(y)$ do not commute). Inserting the perturbative expansions for $\widehat{U}(x),\widehat{U}(y)$ and noting that in general $\langle {\widehat{U}}_{2}(z)\rangle =\langle {\widehat{U}}_{2}^{\dagger }(z)\rangle =2\langle {\widehat{U}}_{1}^{\dagger }(z){\widehat{U}}_{1}(z)\rangle$ for *z* = *x*, *y*, we obtain

85 $$\begin{array}{l}{{\rm{Tr}}}_{{\rm{DF}}}\left[\langle {\Omega }_{{\rm{G}}}| \widehat{\rho }(t)| {\Omega }_{{\rm{G}}}\rangle \right]\\ \qquad=\frac{1}{2}\left(1+\left(1+{L}_{xy}-\left({P}_{x}+{P}_{y}\right)/2\right)\cos (\varphi )\right).\end{array}$$

The visibility *v* is the magnitude of the $\cos (\varphi )$ term, namely

86 $$v=1+{L}_{xy}-\left({P}_{x}+{P}_{y}\right)/2$$

which is Eq. (59) of the main text. The integral forms of *P*_*z*_(*z* = *x*, *y*) and *L*_*xy*_ are given by

87 $${P}_{z}={\lambda }^{2}{\int }_{-\infty }^{+\infty }{\rm{d}}\tau {\rm{d}}{\tau }^{{\prime} }\chi (\tau ){\chi }^{\star }({\tau }^{{\prime} })W(z(\tau ),z({\tau }^{{\prime} })),$$

88 $${L}_{xy}={\lambda }^{2}{\int }_{-\infty }^{+\infty }{\rm{d}}\tau {\rm{d}}{\tau }^{{\prime} }\,\chi (\tau ){\chi }^{\star }({\tau }^{{\prime} })W(x(\tau ),y({\tau }^{{\prime} })),$$

where $\chi (\tau )=\eta (\tau )\exp (-iE\tau )$, and the functions $W(z(\tau ),z({\tau }^{{\prime} })),W(x(\tau ),y({\tau }^{{\prime} }))$ are two-point field correlation functions,

89 $$W(z(\tau ),z({\tau }^{{\prime} }))=\langle \phi | \widehat{\phi }(z(\tau ))\widehat{\phi }(z({\tau }^{{\prime} }))| \phi \rangle$$

90 $$W(x(\tau ),y({\tau }^{{\prime} }))=\langle \phi | \widehat{\phi }(x(\tau ))\widehat{\phi }(y({\tau }^{{\prime} }))| \phi \rangle$$

where $\left|\phi \right\rangle \equiv \left|{\phi }_{{\rm{F}}}\right\rangle$ is the initial state of the field. The field operators in Eq. (89) are pulled back to the worldline of the qubit as parametrised by the coordinates $z(\tau ),z({\tau }^{{\prime} })$, and in the case of Eq. (90), to the two worldlines on different spacetime metrics, $\left|g(x)\right\rangle$, $\left|g(y)\right\rangle$. This calculation straightforwardly generalises to additional detectors, giving a visibility of the form Eq. (60).

### Breaking the Labelling Ambiguity with a Third System

We have shown that for scenarios involving two systems, there exists a generic ambiguity in labelling which system is in superposition, owing to the fact that quantum coordinate transformations can always place one of them in a fixed position while preserving relative distances in each branch. Here, we show that including a third system lifts this ambiguity, relevant to claims about the GIE proposals considered in Superposition of Geometries in the Gravitationally-Induced Entanglement Proposals. Consider a three-particle system in the state,

91 $$\left|\psi \right\rangle =\frac{1}{2\sqrt{2}}(\left|x\right\rangle +\left|{x}^{{\prime} }\right\rangle )\otimes (\left|y\right\rangle +\left|{y}^{{\prime} }\right\rangle )\otimes (\left|z\right\rangle +\left|{z}^{{\prime} }\right\rangle )$$

where ${x}^{{\prime} }=x+X,{y}^{{\prime} }=y+Y,{z}^{{\prime} }=z+Z$ with *X*, *Y*, *Z* fixed superposition sizes for each of the particles. Evolution under the time-evolution operator $\widehat{U}(t)$ and projection onto the joint state $\left|\Omega \right\rangle$ of all three particles, gives,

92 $$\begin{array}{l}\left\langle \Omega |{\hat{U}}(t)|\psi \right\rangle \\ \,\,\,=\frac{1}{2\sqrt{2}}\underbrace{\left\langle \Omega |{\hat{U}}(t)|x\right\rangle \left(\left|y\right\rangle +\left|{y}^{{\prime} }\right\rangle \right)\left(\left|z\right\rangle +\left|{z}^{{\prime} }\right\rangle \right)}_{{A}_{1}}\\\qquad+\frac{1}{2\sqrt{2}}\underbrace{\left\langle \Omega |{\hat{U}}(t)|{x}^{{\prime} }\right\rangle \left(\left|y\right\rangle +\left|{y}^{{\prime}}\right\rangle\right)\left(\left|z\right\rangle +\left|{z}^{{\prime} }\right\rangle \right)}_{{A}_{2}}\end{array}$$

In the case when $\hat{U}(t)$ describes the mutual Newtonian interaction between each pair of particles, this corresponds to a generalization of the GIE scenario in Superposition of Geometries in the Gravitationally-Induced Entanglement Proposals to three particles. We could attempt to classically fix the first particle at position *x* by rewriting the second amplitude in Eq. (92), *A*_2_, as

93 $$\langle {\Omega }_{X}^{{\prime} }| {\widehat{U}}_{X}^{{\prime} }(t)| x\rangle (\left|y-X\right\rangle +\left|{y}^{{\prime} }-X\right\rangle )(\left|z-X\right\rangle +\left|{z}^{{\prime} }-X\right\rangle )$$

that is, the first particle in the localized state $\left|x\right\rangle$ while the other two are jointly shifted by the amount −*X*. Meanwhile, the measurement state is likewise shifted, $\left|\Omega \right\rangle \to \left|{\Omega }_{X}^{{\prime} }\right\rangle \equiv {\widehat{T}}^{\dagger }(X)\left|\Omega \right\rangle$ and the dynamics (on this branch of the superposition) are determined by ${\widehat{U}}^{{\prime} }(t)={\widehat{T}}^{\dagger }(X)\widehat{U}(t)\widehat{T}(X)$. We could now attempt to classically fix the position of the second particle at *y*, which requires rewriting both *A*_1_ and *A*_2_ in terms of the localized state $\left|y\right\rangle$. In particular, *A*_1_ becomes,

94 $$\begin{array}{l}\langle \Omega | \widehat{U}(t)| x\rangle \left|y\right\rangle (\left|z\right\rangle +\left|{z}^{{\prime} }\right\rangle )\\\quad+\langle {\Omega }_{Y}^{{\prime} }| {\widehat{U}}_{Y}^{{\prime} }(t)| x-Y\rangle \left|y\right\rangle (\left|z-Y\right\rangle +\left|{z}^{{\prime} }-Y\right\rangle )\end{array}$$

where we have used the notation $\left|\Omega \right\rangle \to \left|{\Omega }_{Y}^{{\prime} }\right\rangle \equiv {\widehat{T}}^{\dagger }(Y)\left|\Omega \right\rangle$ and ${\widehat{U}}_{Y}^{{\prime} }(t)={\widehat{T}}^{\dagger }(Y)\widehat{U}(t)\widehat{T}(Y)$, while *A*_2_ becomes,

95 $$\begin{array}{l}\langle \Omega | \widehat{U}(t)| x+X\rangle \left|y\right\rangle (\left|z\right\rangle +\left|{z}^{{\prime} }\right\rangle )\\\quad+\langle {\Omega }_{Y}^{{\prime} }| {\widehat{U}}_{Y}^{{\prime} }(t)| x+X-Y\rangle \left|y\right\rangle (\left|z-Y\right\rangle +\left|{z}^{{\prime} }-Y\right\rangle )\end{array}$$

Combining Eqs. (94) and (95) (after normalization) give the full probability amplitude. The key observation here is that the quantum coordinate transformation acts on all considered DoFs, and therefore, one cannot isolate one or more of the subsystems from being affected by such a transformation. Unlike the standard GIE protocol with two particles, with three systems, it is *not* possible to interpret a process (here, involving unitary evolution and measurement in an arbitrary final state) from the perspective of one of the systems being classically fixed while the others undergo modified dynamics.

### Analysis of Setup in Ref. ^61^

Ref. ^61^ considers how quantizing local vacuum fluctuations (either electromagnetic or gravitational) resolves an apparent causality/complementarity paradox. The setup is as follows: an observer, Alice, prepares a particle in a spatial superposition with separation *X*, while Bob has control over a particle at some large distance away, that is initially localized by a trap. At the start of the protocol Bob can decide whether or not to release his particle from the trap, while Alice starts to recombine the paths of her particle. An apparent paradox arises when Alice attempts to measure the coherence of the state of her particle, or Bob seeks to gain which-way information about Alice’s particle (for full details on the causality/complementarity paradox, see Ref. ^61^).

We show below using our framework that whether or not these fluctuations are quantized is independent of the question of whether the metric sourced by Alice’s particle is classical or in quantum superposition. Following the argument in Ref. ^61^, we first assume that Alice’s particle *A* sources the metric field felt by Bob’s particle *B*, in superposition i.e., $(\left|{x}_{{\rm{A}}}\right\rangle \left|g(x)\right\rangle +\left|{x}_{{\rm{A}}}+X\right\rangle \left|g(x+X)\right\rangle )/\sqrt{2}$. Here, $\left|{x}_{{\rm{A}}}\right\rangle ,\left|{x}_{{\rm{A}}}+X\right\rangle$ are position eigenstates of Alice’s particle at *x*, *x* + *X* respectively, and the states $\left|g(x)\right\rangle ,\left|g(x+X)\right\rangle$ live in the metric Hilbert space. The joint state of both particles and the metric sourced by *A* is (see Fig. 1 of Ref. ^61^)

$$\begin{array}{rcl}\left|\psi \right\rangle & = & \frac{1}{\sqrt{2}}(\left|{x}_{{\rm{A}}}\right\rangle \left|g(x)\right\rangle +\left|{x}_{{\rm{A}}}+X\right\rangle \left|g(x+X)\right\rangle )\left|{f}_{{\rm{B}}}(y)\right\rangle \\ & = & \frac{1}{\sqrt{2}}\left|{x}_{{\rm{A}}}\right\rangle \left|g(x)\right\rangle \left|{f}_{{\rm{B}}}(y)\right\rangle +\frac{1}{\sqrt{2}}\left|{x}_{{\rm{A}}}+X\right\rangle \left|g(x+X)\right\rangle \left|{f}_{{\rm{B}}}(y)\right\rangle \end{array}$$

where $\left|{f}_{{\rm{B}}}(y)\right\rangle$ is the state of Bob’s particle (e.g. *f*_B_(*y*) can be a Gaussian wavepacket). Time-evolving the state under a quantum-controlled unitary

$$\widehat{U}=\widehat{U}(x)\otimes \left|{x}_{{\rm{A}}}\right\rangle \langle {x}_{{\rm{A}}}| +\widehat{U}(x+X)\otimes | {x}_{{\rm{A}}}+X\rangle \left\langle {x}_{{\rm{A}}}+X\right|$$

gives

$$\begin{array}{rcl}\widehat{U}\left|\psi \right\rangle & = & \frac{1}{\sqrt{2}}\widehat{U}(x)\left|{x}_{{\rm{A}}}\right\rangle \left|g(x)\right\rangle \left|{f}_{{\rm{B}}}(y)\right\rangle \\ & + & \frac{1}{\sqrt{2}}\widehat{U}(x+X)\left|{x}_{{\rm{A}}}+X\right\rangle \left|g(x+X)\right\rangle \left|{f}_{{\rm{B}}}(y)\right\rangle \\ & = & \frac{1}{\sqrt{2}}\widehat{U}(x)\left|{x}_{{\rm{A}}}\right\rangle \left|g(x)\right\rangle \left|{f}_{{\rm{B}}}(y)\right\rangle \\ & + & \frac{1}{\sqrt{2}}\widehat{U}(x+X){\widehat{T}}^{\dagger }(X)\left|{x}_{{\rm{A}}}\right\rangle \left|g(x)\right\rangle \left|{f}_{{\rm{B}}}(y-X)\right\rangle \end{array}$$

where in the second line we utilize the fact that $\left|x,g(x)\right\rangle ,\left|x+X,g(x+X)\right\rangle$ are related by the unitary translation $\widehat{T(X)}$. The particles are then measured in the position basis $\left|{x}_{{\rm{A}}}^{{\prime} }\right\rangle \left|{y}_{{\rm{B}}}^{{\prime} }\right\rangle$:

$$\begin{array}{l}\left\langle {x}_{{\rm{A}}}^{{\prime} }\right|\langle {y}_{{\rm{B}}}^{{\prime} }| \widehat{U}| \psi \rangle \\ \,\,=\frac{1}{\sqrt{2}}\langle {x}_{{\rm{A}}}^{{\prime} }| \langle {y}_{{\rm{B}}}^{{\prime} }| \widehat{U}(x)| {x}_{{\rm{A}}}\rangle | g(x)\rangle \left|{f}_{{\rm{B}}}(y)\right\rangle \\ \,\,+\frac{1}{\sqrt{2}}\langle {x}_{{\rm{A}}}^{{\prime} }| \langle y{\prime} | \widehat{U}(x+X){\widehat{T}}^{\dagger }(X)| {x}_{{\rm{A}}}\rangle | g(x)\rangle \left|{f}_{{\rm{B}}}(y-X)\right\rangle \\ \,\,=\frac{1}{\sqrt{2}}\langle {x}_{{\rm{A}}}^{{\prime} }| \langle {y}_{{\rm{B}}}^{{\prime} }| \widehat{U}(x)| {x}_{{\rm{A}}}\rangle | g(x)\rangle \left|{f}_{{\rm{B}}}(y)\right\rangle \\ \,\,+\frac{1}{\sqrt{2}}\langle {x}_{{\rm{A}}}^{{\prime} }-X| \langle {y}_{{\rm{B}}}^{{\prime} }-X| \widehat{U}(x)| {x}_{{\rm{A}}}\rangle | g(x)\rangle \left|{f}_{{\rm{B}}}(y-X)\right\rangle \end{array}$$

where to obtain the last line we inserted the identity and used the fact that $\widehat{T}(X)\widehat{U}(x+X){\widehat{T}}^{\dagger }(X)=\widehat{U}(x)$. The last line is an expression of the fact that the setup in Ref. ^61^ can be understood as occurring on a fixed classical metric described by $\left|g(x)\right\rangle$ (which, notably, factors from the above amplitude) while the measurements on the particles *A* and *B* occur in superposition. This, in particular, entails that the position of the “screen” used to measure the position of particle *A* is, in this form of the amplitude, itself in superposition. The key observation from the above is that the same discussion of causality based on “after what time particle *B* can entangle with *A*, depending on their distance” applies here, as we have merely re-described the same scenario. Indeed, we have the exact same distances between the particles as in the original formulation and the same probability amplitudes. The difference is that in this picture, the positions of the particles are not described by independent measurements but appear correlated. Our analysis above for the gravitational case follows analogously to the electromagnetic case, with a replacement of the gravitational field/metric state with the state of the electromagnetic field.

### Dark Matter Scattering

In this section, we highlight the utility of our framework by applying it to another case of decoherence, namely of general relativistic sources of gravity (such as a dark matter clump) by scattering particles. This scenario has been considered recently in the context of dark matter detection^27,28^. The authors of^27,28^ consider, in analogy with Eq. (44), a dark matter (DM) “Schrödinger-cat state” that interacts with its environment $\left|\chi \right\rangle$, leading to an entangled state of the DM and environment:

96 $$\left|\psi \right\rangle =\frac{1}{\sqrt{2}}\left(\left|{{\rm{DM}}}_{1}\right\rangle \left|{\chi }_{1}\right\rangle +\left|{{\rm{DM}}}_{2}\right\rangle \left|{\chi }_{2}\right\rangle \right).$$

The superposition of DM configurations is tacitly defined with respect to some coordinate system in which relative distances between the DM and some incoming particle are expressed. We have demonstrated that the choice of coordinates can change the interpretation of the scenario–here it would be whether decoherence appears in the position of the DM (i.e., the probe particles are in superposition before the interaction) or in the position of the probe particles (i.e., the DM is in superposition before the interaction).

We emphasise that decoherence can be attributed to either of the subsystems involved in the scattering process, implying that it cannot be fundamentally associated with either. Instead, the only physically relevant DoF that decoheres in such a scenario is the relative distance between (in this case) the dark matter and the scattering particles.

The rate of decoherence can be computed through the overlap of the scattering particle states, 〈*ψ*_1_∣*ψ*_2_〉, after interaction with the DM states, $\left|{{\rm{DM}}}_{1}\right\rangle$, $\left|{{\rm{DM}}}_{2}\right\rangle$. The computation of the overlap will in general, depend on the convolution of the wavefunctions of the scattered states, given by^27^,

97 $${S}_{12}=\int \,{{\rm{d}}}^{2}b\int \,{{\rm{d}}}^{3}x\,{\psi }_{s1}^{\star }(t,x){\psi }_{s2}(t,x),$$

where *b* is the impact parameter of the scattering process, while *ψ*_*s*1,2_ denotes the scattered part of the respective wavefunctions. Importantly, Eq. (97) only depends on the wavepacket parameters and the relative distance between the two branches of the DM superposition, which we denote *X*_12_. Taking into consideration the DM distribution and the spacetime generated by it, and the wavefunction of the scattered particle, this means that such a scenario is physically equivalent to that of a single “fixed” DM source from which a particle, in a superposition of relative impact parameters, is scattered. The decoherence in this case is not fundamental to either the scattering particle or the DM; both are equivalent situations that depend only on one’s choice of coordinates.

### Beyond Symmetries of Dynamics

The focus of our analysis has been on superposition states whose components are related by a symmetry of dynamics, such as a translation (or diffeomorphism). More generally, our framework is applicable to scenarios in which the components of a state are not related by a symmetry. First, we know that any two normalized states $\left|{\psi }_{1}\right\rangle ,\left|{\psi }_{2}\right\rangle$ can be transformed into each other by some unitary $\widehat{V}$ e.g., $\left|{\psi }_{2}\right\rangle ={\widehat{V}}^{\dagger }\left|{\psi }_{1}\right\rangle$. When $\widehat{V}$ does not commute with $\widehat{U}$, then in general ${\widehat{U}}^{{\prime} }:=\widehat{V}\widehat{U}{\widehat{V}}^{\dagger }\ne \widehat{U}$. However, the probability amplitudes are still invariant. In particular, for the initial (unnormalised) state $\left|{\psi }_{1}\right\rangle +\left|{\psi }_{2}\right\rangle$, we have the probability amplitude,

98 $$\begin{array}{rcl}\langle \phi | \widehat{U}| {\psi }_{1}\rangle +\langle \phi | \widehat{U}| {\psi }_{2}\rangle & = & \langle \phi | \widehat{U}| {\psi }_{1}\rangle +\langle \phi | \widehat{U}{\widehat{V}}^{\dagger }| {\psi }_{1}\rangle \\ & = & \langle \phi | \widehat{U}| {\psi }_{1}\rangle +\langle \widehat{V}\phi | \widehat{V}\widehat{U}{\widehat{V}}^{\dagger }| {\psi }_{1}\rangle \\ & = & \langle \phi | \widehat{U}| {\psi }_{1}\rangle +\langle \widehat{V}\phi | {\widehat{U}}^{{\prime} }| {\psi }_{1}\rangle \end{array}$$

Thus even states not related by a symmetry can be viewed as relative, but now in some scenarios, the evolution of the whole system can be viewed as occurring “in superposition” under the action of $\widehat{U},{\widehat{U}}^{{\prime} }$.

## Data Availability

No datasets were generated or analysed during the current study.
